# The structure and function of deubiquitinases: lessons from budding yeast

**DOI:** 10.1098/rsob.200279

**Published:** 2020-10-21

**Authors:** Harsha Garadi Suresh, Natasha Pascoe, Brenda Andrews

**Affiliations:** 1 Donnelly Centre for Cellular and Biomolecular Research, University of Toronto, Toronto, Ontario, Canada M5S 3E1; 2 Department of Molecular Genetics, University of Toronto, Toronto, Ontario, Canada M5S 3E1

**Keywords:** deubiquitinases, protein degradation, ubiquitin signalling

## Abstract

Protein ubiquitination is a key post-translational modification that regulates diverse cellular processes in eukaryotic cells. The specificity of ubiquitin (Ub) signalling for different bioprocesses and pathways is dictated by the large variety of mono-ubiquitination and polyubiquitination events, including many possible chain architectures. Deubiquitinases (DUBs) reverse or edit Ub signals with high sophistication and specificity, forming an integral arm of the Ub signalling machinery, thus impinging on fundamental cellular processes including DNA damage repair, gene expression, protein quality control and organellar integrity. In this review, we discuss the many layers of DUB function and regulation, with a focus on insights gained from budding yeast. Our review provides a framework to understand key aspects of DUB biology.

## 1. Introduction

Ubiquitination is a reversible post-translational modification (PTM) that governs a wide variety of cellular processes including protein degradation and sorting, cell signalling and gene expression [1]. Ubiquitination of target substrates occurs through the sequential activity of three enzymes, a ubiquitin (Ub)-activating enzyme (E1), a Ub-conjugating enzyme (E2) and a Ub ligase (E3) [1–5]. This enzyme cascade directs the attachment of Ub chains onto target proteins—the length and specific configuration of Ub chains dictates their functional consequences [1–5]. The ubiquitination state of protein substrates is also determined by deubiquitinases (DUBs) which hydrolyse Ub-substrate and Ub–Ub isopeptide bonds [1–5]. DUBs have three major cellular functions: (i) the generation of free Ub moieties from linear fusions of Ub [1–5]; (ii) trimming of existing polyubiquitin chains; and (iii) reversal of Ub signalling by removal of Ub chains from target proteins [1–5]. Thus, like ubiquitination enzymes, DUBs are master regulators within the Ub system, and impinge on diverse cellular processes. Importantly, mis-regulation of DUB function has been associated with the onset or progression of numerous diseases, including some cancers and Alzheimer's disease [6–9]. Owing to the importance of DUBs in eukaryotic cell biology, there has been a concerted effort to understand their structure, mechanisms of substrate and polyubiquitin chain-type recognition and modes of regulation.

Key insights into DUB biology have emerged from work in model organisms such as the budding yeast*, Saccharomyces cerevisiae.* The yeast model system offers several key advantages for studying DUB function, including a compact genome, the conservation of the proteome among eukaryotes and the ease of maintenance under laboratory conditions [10]. In addition, yeast is highly amenable to a variety of genetic manipulations and exists in stable haploid and diploid states. In this review, we focus on the current state of knowledge about the structure and function of yeast DUBs and discuss the implications of insights gained from work in yeast for understanding DUB function in more complex organisms such as humans.

## 2. Deubiquitinase families in *Saccharomyces cerevisiae*

**Figure 1.**
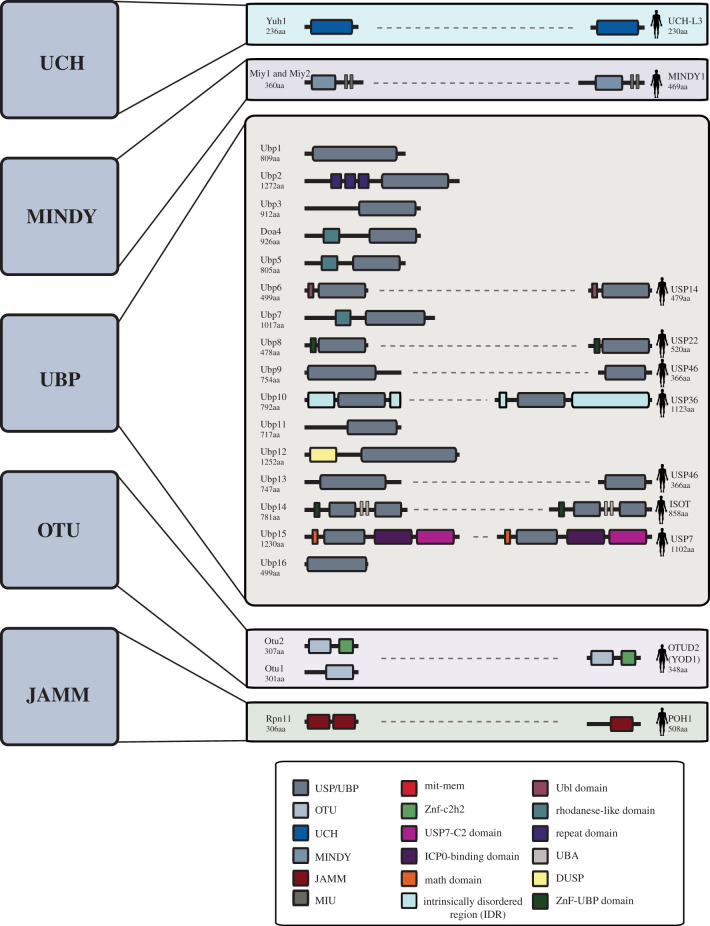
The yeast DUBs. Shown are schematics of the DUB proteins in *S. cerevisiae* categorized by family, illustrating key functional domains (legend at base of the figure). Any human homologues are noted to the right of the protein diagrams. The size of the DUB proteins is indicated on the left of the schematics and domain sizes are indicated according to scale. ‘aa’ refers to amino acids.

The yeast genome encodes 22 putative DUBs that are subdivided into classes based on their sequence and structural similarity. The roster of DUBs encoded by the human genome is more extensive with approximately 100 DUBs that are divided into seven families: the ubiquitin-specific proteases (USP/UBP); ubiquitin C-terminal hydrolases (UCHs); ovarian tumour proteases (OTUs); JAB1/MPN/MOV34s (JAMMs); Josephins, zinc finger with UFM1-specific peptidase domain protein protease (ZUFSP) and the recently discovered MINDY. These families are further subcategorized according to their mode of catalysis: USPs, UCHs, OTUs and MINDYs are cysteine proteases while the JAMMs are metalloproteases. Five of the seven DUB families (USP/UBP, UCH, OTU, JAMM and MINDY) are conserved in yeast (figure 1) [11–13], and below we summarize our understanding of the functions and mechanisms of action of these conserved DUB families.

### 2.1. Ubiquitin C-terminal hydrolases

As noted above, the majority of yeast DUBs, including USPs, UCHs, OTUs and MINDY, are cysteine proteases, which in general have a catalytic triad comprising Cys, His and Asp/Asn residues [5]. The nearby His residue lowers the p*K*_a_ of the catalytic Cys facilitating a nucleophilic attack, with the Asp/Asn residue functioning to stabilize the catalytic His residue [5].

UCHs were the first DUB family to be structurally characterized. The UCH DUBs possess a catalytic domain spanning 230 amino acid residues that adopts a core fold and catalytic triad resembling papain [14]. UCHs frequently contain additional C-terminal extensions that may assist in substrate or target recognition [14–16]. In yeast, Yuh1 is the sole member of the UCH family and closely resembles UCH-L3, a small human DUB (figure 1) [17]. Yuh1 preferentially processes small leaving groups and is inactive against larger Ub fusions [17]. Yuh1 appears to play a key role in the processing of the Ub-like protein Rub1p/NEDD8, a function that is conserved in its human counterpart [17].

### 2.2. Ubiquitin-specific proteases

The USP/UBPs constitute the largest family of DUBs, with 16 members in yeast and 56 members in humans (figure 1 and table 1). USP/UBPs have a core catalytic domain and often possess insertions or terminal extensions that assist in substrate and/or target recognition [1,25,26]. Although the USP/UBP catalytic domains vary considerably in size and sequence, they all adopt a highly conserved structure resembling a right hand with three subdomains, including a finger, palm and thumb [1]. A cleft formed between the palm and thumb subdomains forms the catalytic centre with the thumb and palm containing the catalytic Cys and His, respectively [1], while the finger subdomain interacts with Ub to facilitate its positioning in the catalytic centre. Substrate recognition by USP/UBPs typically involves either their variable sequence regions or scaffolds and substrate adaptors in multi-protein complexes [1]. The overall complexity and diversity seen in the USPs underlies their diverse substrate profile and cellular roles.

**Table 1.** A snapshot of DUBs in budding yeast: localization, post-translational modifications and functions.

DUB	systematic name	localization	site of ubiquitination	site of phosphorylation	functions	references
*UBP1*	*YDL122 W*	ER, cytoplasm	K378, K423, K524	S154, S530, S531, S555, S579, S593, S618, S638, S653	prERAD	[18–22]
*UBP2*	*YOR124C*	cytoplasm	K1065, K980, K991	S614, S876, S901, S907, S917, T875	endocytosis, degradation of misfolded proteins	[18–20]
*UBP3*	*YER151C*	cytoplasm		S173, S242, S243, S329, S329, S335, S339, S341, S360, S364, S400, S663, T106, T337	degradation of misfolded proteins, ribophagy, proteophagy, mitophagy, formation of stress granules, regulation of histone methylation,	[18–22]
*DOA4*	*YDR069C*	cytoplasm, endosome		S414, S443, S498, T500, T502	multi-vesicular body formation, ubiquitin recycling,	[18–20]
*UBP5*	*YER144C*	bud neck		S316, S395	unknown	[18]
*UBP6*	*YFR010 W*	nucleus, cytoplasm	K190, K242, K253, K308, K378	S298, S383, S470, T389	proteasome-mediated protein turover	[18–20,23]
*UBP7*	*YIL156 W*	cell periphery	K549	S155, S193, S229, S235, S486, S488, S852, S863, S868, S924, S925, T120, Y113	endocytosis	[18–20,22]
*UBP8*	*YMR223 W*	nucleus			regulation of transcription	
*UBP9*	*YER098 W*	cytoplasm	K45	S45, S54, S55, S640	unknown	[18,23]
*UBP10*	*YNL186 W*	nucleolus and nucleus		S258, S259, S260, S263, T83	regulation of ribosome biogenesis, chromatin organization and cell cycle	[21,22,24]
*UBP11*	*YKR098C*	mitochondria		S228, S229, S253, S257, T226, T507, T526	unknown	[18,22]
*UBP12*	*YJL197 W*	nucleus and cytoplasm		S1160, S1179, S12, S1204, S1226, S1252, S430, S84, T1153, T1202, T1227, T26, T86	unknown	[18–20]
*UBP13*	*YBL067C*	cytoplasm	K469, K571	S198, S217, S337, S339, S340, S354, S356, S357, S45, S465	unknown	[18–21]
*UBP14*	*YBR058C*	nucleus and cytoplasm	K132, K370, K398, K500, K670, K770		ubiquitin recyling	[18]
*UBP15*	*YMR304 W*	cytoplasm	K1127, K1163, K303, K508, K771		endocytosis and cell cycle	[18]
*UBP16*	*YPL072 W*	mitochondria	unknown	unknown	unknown	
*YUH1*	*YJR099 W*	cytoplasm		S186	unknown	[19]
*OTU1*	*YFL044C*	nucleus and cytoplasm	K267	S134	ER-associated degradation	[18,19]
*OTU2*	*YHL013C*	cytosplasm	K100, K233		unknown	[18]
*RPN11*	*YFR004 W*	nucleus and cytoplasm	K12, K205, K233	S243, T262	proteasome-mediated protein turnover, formation of proteasome storage granules	[18]
*MIY1*	*YPL191C*	cytoplasm	K348, K56	S343	unknown	[18–22]
*MIY2*	*YGL082 W*	cytoplasm	K162, K181, K352	S187	unknown	[18,19]

### 2.3. Ovarian tumour proteases

The OTU family of DUBs were identified based on their homology to the ovarian tumour gene (*OTU*) in fruit flies. In yeast, the OTU DUB family contains two members, Otu1 and Otu2, while in humans, there are 16 (figure 1 and table 1). Crystal structures of yeast Otu1, and several human OTUs, reveal a conserved catalytic OTU domain consisting of five β-strands sandwiched between helical domains that vary in size [1,5,27]. The active site of the OTU domain contains an unusual loop not seen in other thiol-DUBs, and may lack the catalytic Asp/Asn residue, such that several human OTU DUBs are predicted to be catalytically inactive [3,5]. OTUs display remarkable specificity for different poly-Ub-chain linkages. For example, the human OTU, OTUB1 is highly specific for K48-linked chains, while OTUD2 [1,5] shows specificity for atypical Ub chains such as K11, K27 and K33 [28,29]. Some evidence suggests that the substrate and target specificities of OTU DUBs are evolutionarily conserved. For example, the yeast orthologue of OTUD2, Otu1, shares substrate preference and specificity for atypical Ub-chain linkages with OTUD2, and both DUBs function in endoplasmic recticulum-associated protein degradation (ERAD) [28].

### 2.4. MINDY

The recently discovered MINDY family of DUBs is highly conserved, with a core catalytic domain distinct from other DUBs [29]. Structural analysis of the human MINDY-1 in complex with Ub revealed that the C-terminus of Ub sits in the MINDY-1 catalytic groove in a highly conserved hydrophobic pocket. Like many other DUBs, the L73 residue of Ub is required for this interaction and thus catalysis. MINDY-1 has a strong specificity for longer K48-linked Ub chains and prefers to trim Ub from the distal end of chains [30]. Several MINDY DUBs exist in humans, with only two family members in yeast, one of which, Miy1, has demonstrated catalytic activity (figure 1 and table 1). Although Miy1is the yeast orthologue of MINDY-1, it does not discriminate between positions for Ub-chain cleavage and, unlike human MINDY-1, is capable of cleaving shorter Ub chains [30]. The Ub-binding domains of MINDY, motif interacting with Ub (MIU), are responsible for MINDY chain preference and are discussed in further detail below.

### 2.5. JAB1/MPN/MOV34 metalloenzymes (JAMM/MPN±)

JAMM DUBs are conserved metalloproteases with a Glu-x [N]-His-x-His-x[10]-Asp motif which coordinates binding of two Zn^2+^ ions [1,3]. Catalysis requires a nucleophilic attack by the DUB on the carbonyl carbon of the isopeptide bond in the Ub chain, mediated by an activated water molecule [3]. Rpn11, a proteasome-associated DUB, is the sole representative of the JAMM DUBS in yeast [31,32] (figure 1 and table 1). By contrast, the human genome encodes 14 putative JAMM DUBs.

## 3. Substrate recognition

**Figure 2.**
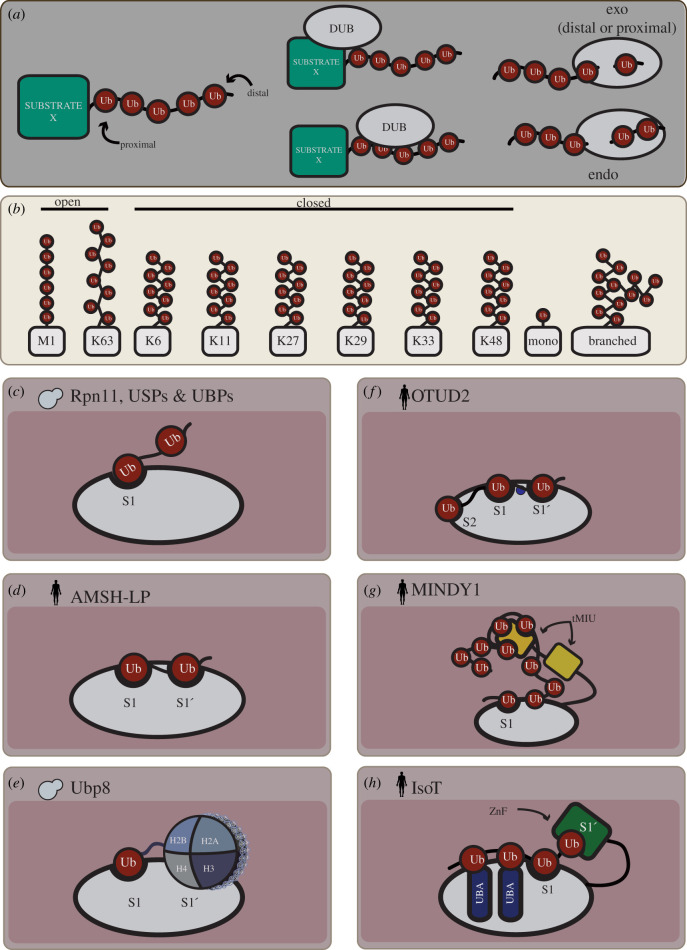
Mechanisms of substrate recognition by DUBs. (*a*) Overview of DUB recognition of Ub chains. A generic substrate ‘X’ with an attached Ub chain is illustrated on the left, with the proximal Ub being substrate bound, while the distal Ub is unmodified. The cartoons in the middle of the panel show that DUBs can recognize their substrates either through interaction with a specific target protein that they deubiquitinate or through interactions with the Ub chain itself. Schematics on the right illustrate endo or exo-cleavage activity associated with DUBs (the exo activity can originate at the distal or proximal end of a Ub chain). (*b*) Types of Ub chain linkages. The various types of Ub chain linkages are illustrated. The M1 and K63 chains, which adopt open conformations, and K6, K11, K27, K29, K33 and K48-linked chains, which adopt closed conformations, are grouped together. Mono-ubiquitination and branched Ub chains may also occur. (*c*–*h*) DUB mechanisms for substrate recognition. (*c*) Ub–S1 site interaction. DUBs that show little or no specificity for Ub chain linkages, such as Rpn11 and several USP/Ubp DUBs, contact Ub through an S1 site only. (*d*) Ub–S1–S1′ interactions. DUBs may contact Ub through both the S1 site and an S1′ site which can impart additional linkage specificity. For example, the RPN11-related DUB AMSH-LP achieves linkage specificity for K11-linked Ub chains through contacts made by its S1′ site. (*e*) S1′ site interaction with target. DUBs such as Ubp8 contain an S1′ site that recognizes a target protein, an interaction that does not involve Ub. (*f*) Ub–S1–S1′–S2 interactions. OTUD2 is an example of a DUB with an S2 domain in its catalytic site, which can accommodate longer K11-linked Ub chains. (*g*) DUB–Ub interactions outside of catalytic domain. Some DUBs such as MINDY-1 contain Ub-binding sites outside of their catalytic domain. The diagram illustrates the case of MINDY-2, in which tandem MIU (tMIU) domains work together to mediate the recognition of longer K48-linked Ub chains. (*h*) Multiple Ub-binding domains. IsoT achieves substrate specificity for K48-linked chains through several interactions with Ub chains using its four Ub-binding domains: ZnF-UBP, USP/UBP domain and two UBA domains, which are inserted into its catalytic domain.

DUBs select their targets through binding to a protein substrate that they deubiquitinate, enabling regulation of specific cellular pathways and processes, or by direct recognition of select Ub-chain types, allowing control of the abundance of distinct Ub linkages (figure 2). For example, several DUBs in the UBP/USP family bind target proteins through additional protein–protein interaction (PPI) domains and will cleave an Ub chain from a specific target regardless of chain architecture [33,34]. By contrast, other DUBs, as discussed in more detail below, display remarkable chain specificity and in some cases will cleave only one linkage type [28,29,33,34].

### 3.1. Linkage specificity

Ubiquitination is a highly versatile PTM because it occurs as eight distinct types of multi-Ub chains, which convey unique structural and functional information. To form multi-Ub chains, Ub moieties are linked to the C-terminal Gly of another Ub protein via one of seven Lys residues (Lys6, Lys11, Lys27, Lys29, Lys33, Lys48, Lys63) or the amino terminus (Met1) (figure 2*b*) [35]. Poly-Ub chains have specific conformational and dynamic properties that are recognized by distinct Ub-binding domains; for example, K48, K6, K11 and K29-linked Ub chains are generally thought to adopt a compact conformation, while M1 and K63-linked Ub chains have an open conformation (figure 2*b*) [35]. These varied linkages help determine specific functions for Ub chains within the cell, including roles for the typical Lys48- and Lys63-linked chains in protein degradation and cell signalling respectively, with atypical chain linkages more likely to be involved in a wide variety of cellular processes including cell cycle regulation and transcription, among others [35].

A significant factor in the diversity of Ub-chain function reflects the selectivity of DUBs for Ub chains of varying lengths and linkages. For example, in yeast, the Ubp8 DUB strongly prefers the mono-ubiquitinated histone component, H2B, Ubp12 and Ubp15 favour K48-linked Ub chains [36–39], while the proteasome-associated Rpn11 DUB, shows little substrate preference [31,32]. All DUBs contact Ub through at least one Ub-binding site called the S1 site in the catalytic domain (figure 2*c–h*). The S1 site is considered a major determinant of DUB specificity as it contacts 20–40% of Ub's surface and contributes significantly towards correct positioning of the Ub C-terminal tail in the DUB's catalytic core [3]. Other Ub contacts may occur through the so-called S1′ site of some DUBs, which further aids in correctly orienting the modified Lys or Met residue towards the catalytic centre (see below, figure 2*c*) [33,34]. Other Ub-binding sites (S2, S2′, S3, S3′ etc.) may also be present in the catalytic domain or on other Ub-binding domains in specific DUBs. Together, these diverse Ub-binding sites contribute to the mode of binding of an individual DUB to its substrate and as such its specificity [33,34].

### 3.2. Substrate specificity via the catalytic domain

One simple mechanism for DUB substrate specificity involves interactions with the proximal Ub on the target substrate, which determines the Lys linkage presented to the catalytic centre. This mechanism is beautifully illustrated by structural studies of Rpn11, a proteasomal-associated DUB that displays promiscuity towards Ub chains, and a related DUB, AMSH-LP, which displays specificity to K63-linked di-Ub chains (figure 2*c,d*) [31,32,40]. AMSH-LP forms extensive contacts with residues that neighbour K63 in the proximal Ub [40] only when K63 linkage occurs, thus ensuring that AMSH-LP is specific to K63-linked Ub chains (figure 2) [40]. Rpn11 does not contact the proximal Ub and instead appears to contact the distal Ub exclusively (figure 2) [31,32], a property that is thought to contribute to its lack of substrate specificity [31,32,40].

### 3.3. Substrate specificity via ubiquitin-binding domains

As previously mentioned, DUBs can contact Ub through sites outside of their catalytic domain, and we discuss key examples of this class of DUBs below.

#### 3.3.1. Ubp14/IsoT

The Ubp14 DUB disassembles unanchored poly-Ub and recognizes K29, K48 and K63-linked Ub chains. The loss of Ubp14 function in *S. cerevisiae* results in defects in proteasomal degradation and the accumulation of unanchored Ub. While the mechanism of action of Ubp14 remains poorly understood, its general function and substrate preference is conserved in its human orthologue, IsoT, which has been studied extensively [41–44]. IsoT contains four putative Ub-binding domains, a ZnF-UBP domain, a USP/UBP domain and two UBA domains, which are inserted into the catalytic domain, and each of these domains form extensive interactions with different Ubs in a Ub chain [45]. The ZnF-UBP contains the S1′ site and as such recognizes the C-terminus of the proximal Ub, while the S1 pocket in the USP/UBP, the UBA1 and the UBA2 domains recognize the second, third and fourth Ubs in the chain, respectively (figure 2*h*) [45]. Interestingly, it appears that the ZnF-UBP and UBP domain enable discrimination of K48-linked Ub chains, while the UBA domains cannot [44,45]. Taken together, these studies offer insight into how a DUB can use different Ub-binding domains to determine substrate specificity.

#### 3.3.2. MINDY-1

Members of the MINDY family of DUBs are thought to recognize Ub chains through tandem MIU motifs (tMIU) that function cooperatively to mediate substrate recognition. Like the Ubp14 example above, the substrate specificity of the yeast MINDY DUB, MIY1, remains unexplored relative to its human orthologue, MINDY-1, which specifically binds K48-linked Ub chains with a preference for longer chains [30,46]. MINDY-1 contains two tandem MIU domains, tMIU1 and tMIU2, that are separated by a linker sequence (figure 2) [30,46]. tMIU2 imparts substrate specificity to MINDY-1 by interacting with three Ub moieties in a K48-linked chain through distinct side chains [46], while tMIU1 has no poly-Ub-binding capacity but instead appears to enhance the affinity of tMIU2 domain for Ub [46]. Recent crystal structures of the MINDY-1 tMIUs in complex with a cyclic K48-tetra Ub chain revealed that the K48-tetra Ub is wrapped around two helices [46]. In this arrangement, a single tMIU2 makes simultaneous interactions with three Ub moieties through three distinct binding sites (figure 2) [46]. The first is formed through hydrophobic interactions and hydrogen bonds between tMIU2 and the middle Ub in the Ub chain [46]. The second occurs between tMIU2 and the proximal Ub and is mediated through hydrophobic interaction between tMIU2 and the I44 hydrophobic patch of Ub. However, this interaction strategy may not be broadly used, as the residue mediating this interaction is poorly conserved among the tMIUs [46]. The third binding interface is formed between tMIU2 and the hydrophobic patch of the distal Ub [46]. The MINDY-1 structure further revealed that tMIU2 binds Ub in an orientation that is exhibited only by longer K48-linked Ub chains, thus explaining its linkage specificity (figure 2).

Taken together, the mechanism of substrate recognition by MINDY-1 demonstrates how Ub-binding domains can work in together in tandem to achieve substrate recognition and specificity [46].

#### 3.3.3. Otu1/OTUD2

As previously discussed, the OTU DUBs display remarkable specificity for their target substrates. The two yeast OTU DUBs, Otu1 and Otu2, are both orthologues of human OTUD2. Otu1 cleaves atypical Ub linkages (K11, K27, K29 and K33), as well as K48-linked Ub chains [28]. Unlike the examples discussed above, crystal structures of Otu1 failed to reveal the mechanism by which Ub-linkage specificity is achieved, although other evidence suggests that Otu1 substrate specificity may be mediated through interactions with one of its targets, Cdc48, and its associated adaptors [28].

Like Otu1, human OTUD2 also cleaves atypical Ub linkages with a preference for longer K11 Ub chains, but differs from Otu1 in that it fails to act on K48 linkages [28]. OTUD2 contacts Ub chains using its S1 and S1′ sites which are located in its catalytic domain. The S1′ site appears to be particularly important for the activity of OTUD2 on K11 linkages, as mutations in this region abolish the K11 linkage specificity of the enzyme (figure 2) [28]. OTUD2 additionally binds Ub chains using an S2 site in its catalytic domain, in an orientation that favours longer K11 Ub chains (figure 2) [28]. While this site is conserved in higher eukaryotes, it is not conserved in *S. cerevisiae*, and whether Otu1 uses a similar mechanism to achieve substrate specificity remains to be determined [28].

## 4. Regulation of deubiquitinase activity

Because DUBs have a wide range of roles in the cell, a series of different regulatory mechanisms have evolved to tightly control DUB activity. In this section, we discuss how PTMs, regulatory domains and PPIs regulate DUB activity in yeast.

### 4.1. Substrate-mediated activation

Several DUBs have evolved allosteric mechanisms to regulate their activity. For example, structural analysis of UCH-L3, the human orthologue of the yeast DUB Yuh1, revealed that the active site undergoes significant conformational changes upon Ub binding. When unbound, UCH-L3 contains a disordered loop that covers its active site, which is stabilized upon Ub binding into an α-helix which forms contacts with Ub. This loop is conserved in several DUBs including Yuh1 [44,45], suggesting that a similar allosteric mechanism may be a feature of a subset of DUBs. Comparisons of Ub-bound Otu1 to the OTU domain of human DUB Otubain 2 alone revealed a disordered loop, which is positioned to sterically clash with a bound Ub. In Ub-bound Otu1, this region forms an ordered β-strand, suggesting that Ub binding to Otu1 may induce a conformation change that stabilizes the disordered region in a β-strand, removing this steric hindrance [47].

Another common mechanism of DUB regulation involves the catalytic residues of DUBs adopting a non-active state. For example, binding of Ub to MINDY-1, the human homologue of Miy1, induces the movement of its Cys loop and allows the catalytic residues to adopt an active conformation. When not bound to Ub, the catalytic His residue in MINDY is flipped away from the catalytic Cys residue, producing an inactive state [30]. Whether this mechanism is conserved in Miy1 is currently unknown.

### 4.2. Complex-mediated activation

Several DUBs are activated through complex binding, including the yeast DUBs Ubp8 and Ubp6. Ubp8 is part of the histone-modifying complex, SAGA, and is specifically responsible for the deubiquitination of mono-ubiquitinated histone component H2B chains [35–38]. Free Ubp8 is generally in an inactive state and activation is induced through interactions with three SAGA complex components, Sgf73, Sgf11 and Sus1. Sgf11 and Sgf73 use their ZnF domains to interact with two distinct regions of the catalytic domain of Ubp8 to promote an active catalytic core, while Sus1 functions to stabilize interactions involving Ubp8, Sgf11 and Sgf73 [35–38]. Strikingly, Sgf11 forms part of the Ubp8 catalytic lobe, creating a highly conserved extended interface with the Ubp8 catalytic domain that aides in target and substrate recognition [35–38]. Crystal structures of Ubp8 bound to a mono-ubiquitinated nucleosome suggest that Ubp8 may have evolved a histone-specific S1′ site in its catalytic domain [37] (figure 2*e*). The Ubp8 complex with mono-ubiquitinated nucleosomes provides one of the first views of a DUB-non-Ub substrate complex and may serve as a model for how other mono-Ub targeting DUBs might recognize their substrates.

Ubp6 is a proteasome-associated DUB, whose activity is dramatically increased upon binding to the 26S proteasome [26]. Structural analysis of free Ubp6 revealed two surface loops blocking access to the catalytic site [26], leading to the hypothesis that binding to the proteasomal component Rpt1 induces an active conformational state by relieving this steric hindrance [26]. Human proteasome-associated DUBs, UCH37, USP14 and POH1, all demonstrate similar proteasome-induced activation.

## 5. Deubiquitinase functions in protein turnover and ubiquitin homeostasis

**Figure 3.**
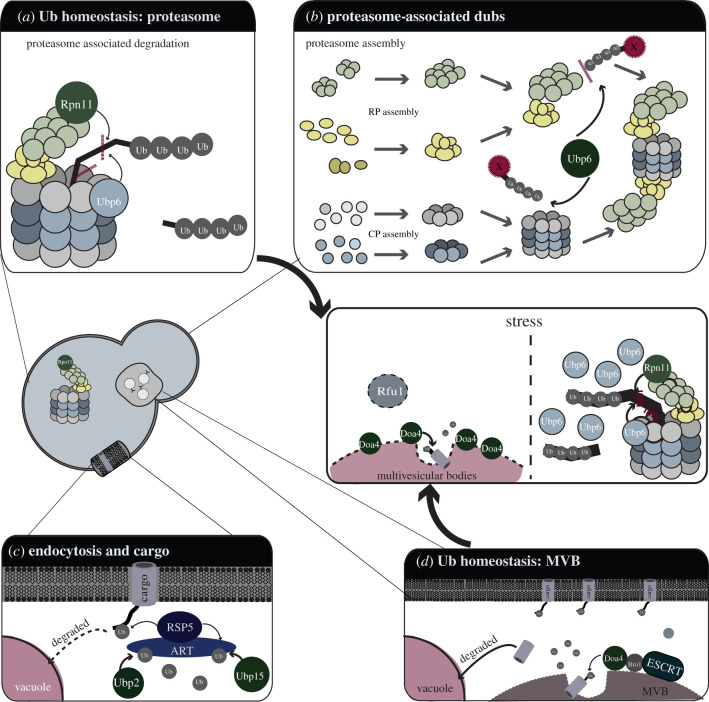
Functions for DUBs in protein turnover and Ub homeostasis. (*a*,*d*) DUB roles in Ub homeostasis. The diagrams show the roles for the DUBs Doa4 (*d*), Ubp6 and Rpn11 (*a*) in ubiquitin homeostasis. Doa4 functions to recycle Ub from ubiquitinated substrates that are targeted to the vacuole. Ubp6 and Rpn11 are both associated with the proteasome and remove Ub from proteins targeted for proteasomal degradation. The inset panel illustrates upregulation of Ubp6 and Doa4 to increase Ub recycling during periods of stress. An inhibitor of Doa4, Rfu1, is downregulated in response to stress, enhancing the activity of Doa4. (*b*) DUBs in proteasome assembly. The key role for Ubp6 in proteasome assembly is illustrated. During proteasome assembly, components of the proteasome (particularly the RP with several Ub-binding domains/motifs) can interact with ubiquitinated substrates, hindering the formation of the complete proteasome. Ubp6 functions to remove Ub from substrates during the assembly process, facilitating maturation of the proteasome. (*c*) DUB roles in endocytosis and cargo turnover. The diagram illustrates the function of the ART complex and Rsp5 Ub ligase in ubiquitination of defective membrane proteins, (cargo) targeting them for degradation in the vacuole. The Ubp2 and Ubp15 DUBs positively regulate ART/Rsp5 by deubiquitinating the complex, thus contributing to cargo turnover and the maintenance of plasma membrane integrity.

Selective spatio-temporal regulation of unwanted or damaged protein is central to cellular homeostasis, adaptation to stress conditions and development of multicellular organisms. Cells have evolved multiple Ub-dependent machineries and processes for protein degradation, including the proteasome, selective autophagy and endocytosis. Below, we review how DUBs function within these proteostasis bioprocesses (summarized in figure 3).

### 5.1. Proteasome-associated deubiquitinases

The proteasome is an approximately 2.5 MDa protein complex consisting of two large subcomplexes, a barrel-shaped core particle (CP) and the regulatory particle (RP) that binds to the axial end of the CP. The RP in turn comprises two subcomplexes of its own, the lid and the base [48], which is proximal to the CP and contains six ATPases that form a ring structure that abuts the CP (figure 3*b*). The proteolytic active sites of the proteasome are buried within the barrel-shaped CP [49], with access restricted by narrow gates at either end of the CP, which are modulated by the Rpt2 subunit of the base [50]. While this ‘restricted access’ arrangement may help prevent the unregulated destruction of intracellular proteins, it also imposes a requirement for substrate unfolding to pass through the narrow opening of the translocation channel leading to the CP, effected by the six ATPases of the base [51]. The Ub chain(s) on substrates being targeted for proteasomal degradation impose a steric constraint preventing translocation through the narrow opening into the catalytic core and must be removed to facilitate substrate degradation. Two DUBs, Rpn11 and Ubp6, are responsible for DUB activity at the proteasome [52–59] and have an interesting functional interplay. Mutation of *RPN11* causes decreased rates of substrate degradation, while the loss of *UBP6* is reported to result in the stabilization of some substrates and destabilization of others, thus indicating distinct and shared function at the proteasome [50,53–57,60–62]. Ubp6 and its human orthologue Usp14 was recently shown to interact preferentially on Ub–cyclin B conjugates with multiple sites of ubiquitination in a chain type-independent fashion and this might explain the differential effects of Ubp6 on substrate degradation [59]. Ubp6 non-catalytically and allosterically affects the rates of proteasomal degradation by interfering with critical substrate deubiquitination by Rpn11, stimulating 20S gate opening, thus increasing access to the degradation chamber and enhancing the rates of ATP hydrolysis by the base ring [58,60,63,64]. While Ubp6 was believed to trim Ub chains at the proteasome, recent evidence indicates that like Rpn11, Ubp6 is also able to cleave Ub chains *en bloc* [59,60] (figure 3*a*).

Ubp6 and Rpn11 also play a key role in the biogenesis and stability of the RP subcomplex of the proteasome [65,66]. RP biogenesis is an elaborate process involving dedicated chaperones [67]. However ubiquitinated substrates, with or without substrate shuttling factors, compete with RP subunits for interaction surfaces on precursor RPs that are en route to form a functional complex [66]. The deubiquitinating activity of proteasome- associated Ubp6 (and perhaps other DUBs) destabilizes such unproductive interactions thus promoting proteasome maturation [66] (figure 3*b*).

### 5.2. Deubiquitinase-mediated regulation of the core endocytic machinery and cargo turnover

Ub-dependent sorting of plasma membrane (PM) proteins is an important and conserved regulatory mechanism. In yeast, the Rsp5 E3 ligase targets PM proteins for ubiquitination (mono-ubiquitination or K63-linked polyubiquitination) and subsequent internalization by endocytosis (figure 3*c*). The resulting vesicles containing ubiquitinated proteins then mature into multi-vesicular bodies before their contents are degraded after fusion with the vacuole [68]. Endocytosis requires the coordinated actions of more than 60 different proteins at the PM [69,70] and several core endocytic adaptors, including Ent1, Ent2, Ede1 and Sla1, harbour Ub-binding domains or interaction motifs [71] and many others are ubiquitinated by the Nedd4-family ligase, Rsp5 [72–76]. Two DUBs, Ubp7 and Ubp2, deubiquitinate Ede1, which is functionally important as *ubp2Δubp7*Δ cells exhibit extended endocytic coat lifetimes and abnormal trafficking of endocytic proteins to early endosomes. Thus, DUBs facilitate initiation of endocytosis and subsequent recruitment of the endocytic coat [77].

The Rsp5 ubiquitin ligase also targets defective PM proteins for degradation [78,79], and promotes the downregulation of properly folded transporters and receptors in response to specific biological stimuli. A family of adaptor proteins termed arrestin-related trafficking adaptors (ART) target specific PM proteins by recruiting Rsp5, which modifies both the cargoes and the ARTs (figure 3*c*). Thus, ARTs and Rsp5 together provide a cargo-specific quality control pathway that mediates endocytic downregulation of diverse PM proteins in response to specific stimuli [80], maintaining the integrity of PMs.

The Ubp2 DUB interacts with Rsp5 via the adaptor protein Rup1 [81], to regulate Rsp5 activity at multiple levels. First, Ubp2 positively regulates Rsp5-mediated ubiquitin signalling by removing the inhibitory autoubiquitination of Rsp5 [82]. Second, Ubp2, together with Ubp15, deubiquitinates ARTs, thus preventing their degradation via the proteasome (figure 3*c*). Third, Ubp2 deubiquinates Rps5 substrates at the PM and thereby antagonizes the degradation of Rsp5 substrates both *in vivo* and *in vitro* [81,83,84]. Which of the opposing effects that Ubp2 has on Rsp5 activity dominates to determine whether a substrate is degraded is probably substrate-dependent and modulated by other cellular factors or stress conditions.

### 5.3. Deubiquitinase regulation of multi-vesicular body formation and function in ubiquitin homeostasis

Ubiquitinated PM protein cargoes are processed through the endocytic pathway through a complex system that involves the ESCRT 0-III complexes which bind and sort ubiquitinated substrates into multi-vesicular bodies (MVBs) that ultimately fuse with the vacuole [85]. However, prior to being engulfed in the MVBs, Bro1, an ESCRT III adaptor protein, recruits the DUB Doa4 to endosomes, and stimulates its enzymatic activity resulting in deubiquitination of the targets aiding in the formation of MVBs [86–89]. MVBs in turn fuse with the vacuole to empty their contents and facilitate degradation of their cargo in the vacuole [86–89] (figure 3*d*). This example illustrates a key role for a DUB in determining the cargoes of the MVBs and thereby their degradation.

The endosome-associated Doa4 DUB discussed above, as well as the proteasome-associated DUBs Ubp6 and Rpn11, also have major roles in trimming and/or *en bloc* removal of Ub chains from target substrates, thereby promoting the recycling of Ub [90,91]. In logarithmically growing cells, Ub exists in a mixed population of: (i) monomers; (ii) unanchored chains; and (iii) anchored chains of different linkages on substrates [3,92]. Given the short half-life of Ub (2 h) [93], recycling of Ub from unanchored and anchored polyubiquitin chains is key for restoring the ubiquitination capacity of cells, particularly under stress conditions such as heat shock [41,54,90]. Ub depletion leads to specific transcriptional upregulation of *UBP6* as a compensatory mechanism to replenish Ub levels by increasing the proteasomal capacity to deubiquitinate its substrates prior to degradation [94]. Similarly, under heat shock conditions that necessitate increased Ub recycling, *DOA4* transcription is upregulated. Doa4 activity is further enhanced by selective downregulation of a Doa4 inhibitor, Rfu1, both at the transcriptional level and at the protein level by Rsp5-mediated selective degradation by the proteasome (figure 3, inset) [87,90,95]. Thus, the Ubp6 and Doa4 DUBs regulate Ub recycling using a highly coordinated and regulated circuitry (figure 3, inset). Other DUBs also contribute to Ub homeostasis. In particular, Ubp14 cleaves unanchored polyubiquitin chains to replenish cells with monomers of Ub [41], while Ubp3, Ubp8 and Ubp10 contribute to recycling of ubiquitin from polyubiquitin chains, though the significance of their action in Ub homeostasis remains poorly understood [90,96].

## 6. Deubiquitinase-mediated regulation of protein quality control

As discussed above, proteotoxic stress caused by heat or other environmental insults induces global protein unfolding/misfolding [97] which causes a profound and multi-component cellular response. First, quality control systems mediated by chaperones and the Ub proteasome or autophagy systems are triggered, to either refold or degrade toxic non-native species. Alternatively, proteins may remain in an unfolded state, and accumulate as protein aggregates at discrete sites in the cell [98–102]. Finally, as a compensatory mechanism, translation is shut down upon severe heat shock to prevent accumulation of additional misfolded species [103–105]. Thus, living systems have evolved elaborate mechanisms for protein quality control (PQC), and DUBs contribute to these mechanisms in several ways, as outlined below.

### 6.1. Regulation of cytosolic protein quality control

**Figure 4.**
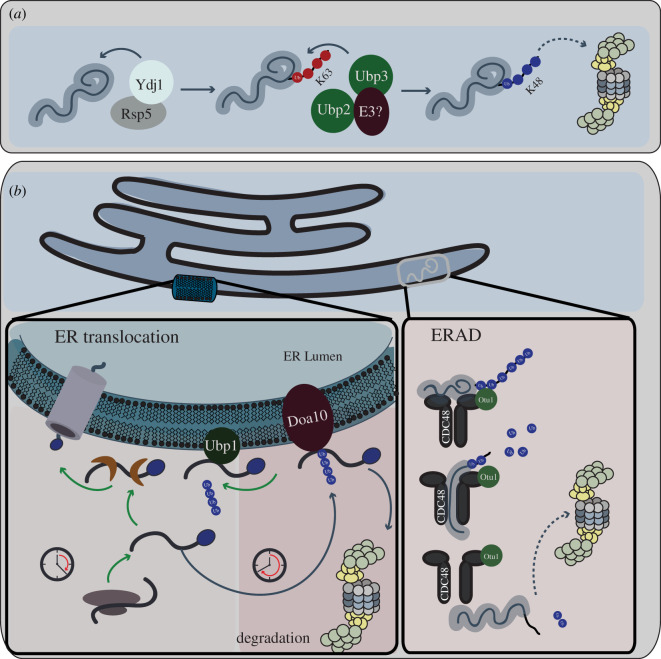
DUB functions in protein quality control. (*a*) DUB roles in regulation of misfolded proteins. The diagram illustrates targeting of misfolded proteins to the proteasome by the DUBs Ubp2 and Ubp3. Misfolded proteins are ubiquitinated by the Ydj1–Rsp5 complex, resulting in the addition of K63-linked Ub chains. As proteasomal targeting requires K48-linked Ub chains, Ubp2/Ubp3 and an unknown E3 ligase function to remove these K63-linked chains and remodel to K48-linked chains. The misfolded proteins are then degraded by the proteasome. (*b*) Role for DUBs in ER-associated protein homeostasis. The left panel illustrates the interplay of the ER-associated E3 ligase Doa10 and DUB Ubp1 in the pre-insertional ER associated degradation (prERAD) response. By recognizing C-terminal hydrophobic motifs (blue circle on peptide), prERAD tags pre-inserted proteins for degradation that have remained on the cytosolic leaflet of the ER for too long. If proteins that have a GPI-anchor sequence fail to translocate to the ER in a timely manner, they are ubiquitinated by the E3 ligase Doa10. Molecular chaperones (brown crescents) aid in both translocation of proteins into the ER and targeting of non-inserted GPI-anchored proteins to Doa10-mediated ubiquitination and subsequent degradation. Ubp1-mediated deubiquitination allows these ubiquitinated substrates to avoid immediate degradation and as such promotes translocation into the ER. However, if their translocation remains unsuccessful then Doa10-mediated degradation dominates to degrade the prERAD substrates. Thus, Ubp1 acts as a ‘molecular timer’ deciding the fate of prERAD substrates. The right panel diagrams the function of the Otu1 DUB in ER-associated degradation (ERAD) of substrates that are polyubiquitinated by Hrd1. The Cdc48 ATPase (black shape) is recruited to the ER membrane and uses ATP hydrolysis to pull the polypeptide substrate out of the membrane. The complex of Cdc48 ATPase and substrate leaves the membrane, and the DUB Otu1 trims the Ub chain, allowing release of the substrate from Cdc48, and its subsequent degradation by the proteasome.

Upon proteasomal inhibition, misfolded proteins tend to partition into two compartments—the juxta/intra-nuclear quality control compartment (JUNQ/INQ), and the insoluble protein deposit (IPOD) that is situated near the vacuole and colocalizes with the pre-autophagosomal structure [106–108]. A variety of studies implicate DUBs in PQC involving these compartments. First, over-expression of Doa4 causes partitioning of a reporter for misfolded proteins, Ubc9ts-GFP, to the IPOD upon proteasomal inhibition, while the reporter normally localizes to both the JUNQ/INQ and IPOD compartments in wild-type cells [106]. This phenotype may reflect the direct action of Doa4 on the misfolded model substrate or may be an indirect consequence of the role of Doa4 in Ub recycling. Second, overproduction of Ubp3 also positively regulates the degradation of Ubc9ts-GFP and can offset a deficiency in Hsp70 chaperone activity both during heat shock and ageing [109]. Ubp3 and its cofactor Bre5 are recruited to heat-induced protein aggregate–stress granule complexes [103,104] and stationary phase-induced stress granules [110,111], consistent with a direct role of Ubp3 in quality control of functional and misfolded aggregates. Third, the E3 ligase Rsp5, with assistance from the Hsp70 co-chaperone Ydj1, recognizes interaction motifs in misfolded proteins upon severe heat shock that are otherwise hidden resulting in their K63 polyubiquitination [112]. However, K63-polyubiquitinated substrates are poorly recognized by the proteasome and thereby not efficiently degraded [113]. Ubp3, along with Ubp2, associates with Rsp5 to reverse the K63 polyubiquitin chains that Rsp5 builds on its misfolded substrates and instead promotes K48 polyubiquitination by an unknown E3 ligase, targeting them for proteasomal degradation [114]. Thus, the chain editing functions of DUBs can facilitate the degradation of misfolded proteins (figure 4*a*), in collaboration with other enzymes. The mechanism of recognition of the severity of protein aggregation/damage by DUBs, and the interplay with the Hsp70/Hsp40/Hsp104 tri-chaperone system and the proteasome, remains largely unexplored.

### 6.2. Regulation of quality control of tail-anchored proteins

While misfolded proteins represent a major class of PQC substrates, a substantial fraction of the secretome transiently resides in the cytosol before translocating into the ER, and represents another class of substrates that needs to be controlled by the proteostatic machinery. Cells have evolved a mechanism termed pre-insertional endoplasmic reticulum-associated degradation that involves recognition of the C-terminal hydrophobic glycosylphosphatidylinositol (GPI) anchoring sequences of non-inserted tail-anchored proteins that have a prolonged residence time on the cytosolic leaflet of the ER by the Doa10 E3 ligase, which targets them for degradation via the proteasome [115]. Ubp1, an ER-associated DUB [116], antagonizes the activity of Doa10 by deubiquitinating its substrates and thus extending their residence time at the cytosolic leaflet of the ER [115]. Thus, the interplay between Doa10 and Ubp1 acts as a ‘timer’ determining the fate of secreted proteins (figure 4*b*, left panel).

### 6.3. Regulation of endoplasmic reticulum-associated protein degradation

Protein homeostasis in the ER is maintained by a quality control system called the ERAD pathway that retains misfolded proteins in the ER, and promotes their ultimate retrotranslocation into the cytosol, where they are polyubiquitinated, and degraded by the proteasome [117,118]. The recognition of a misfolded protein in the ER lumen is best understood for misfolded glycoproteins [117,119]. The N-linked glycan on these proteins is trimmed to generate α-1,6-mannose residue that is recognized by the multi-subunit HRD complex, which includes the Hrd1 ubiquitin protein ligase. Once a segment of the substrate reaches the cytoplasmic side of the ER membrane, it is polyubiquitinated by Hrd1 in concert with the E2, Ubc7 and its activator Cue1 [118]. A complex containing the Cdc48 ATPase is recruited to the Hrd1 complex via recognition of the polyubiquitin chain by its cofactor Ufd1/Npl4, and the polyubiquitinated substrate is then pulled through the central pore of Cdc48. The DUB Otu1 interacts with Cdc48 to trim the polyubiquitin chain (but does not completely remove the chain) facilitating further threading of the substrate through the central pore of Cdc48. Thus, Otu1 assists Cdc48 in the extraction of misfolded proteins across the ER membrane and targets the substrates that continue to be ubiquitinated for proteasomal degradation [120]. The relationship between Otu1 and Cdc48 provides another illustration of how DUB-mediated polyubiquitin chain editing plays a crucial regulatory role in coordinating protein turnover (figure 4*b*, right panel).

## 7. Deubiquitinase-mediated regulation of ribophagy and proteaphagy

**Figure 5.**
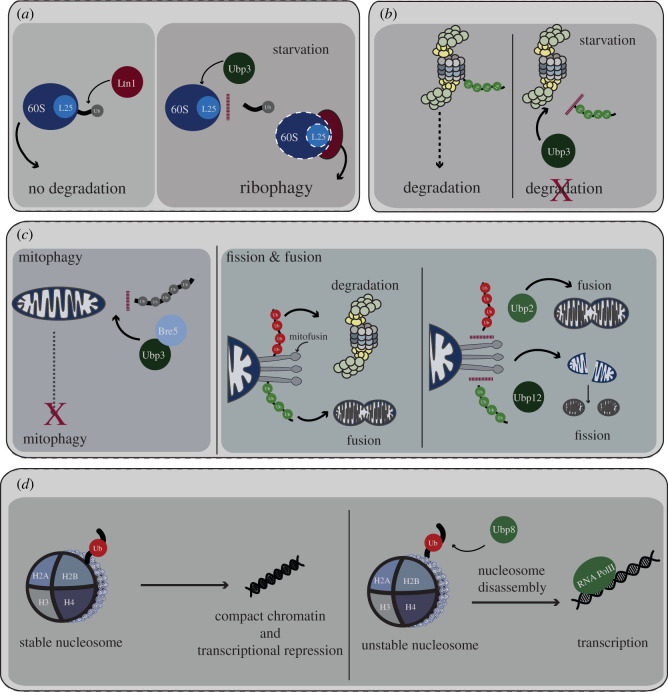
DUB roles in the degradation of protein complexes. (*a*) Ribophagy. Regulation of ribophagy by the Ubp3 DUB under conditions of starvation is illustrated. Ribophagy involves ubiquitination of the 60S ribosomal protein subunit Rpl25 (L25) by the E3 ligase Ltn1. Ubiquitination acts as a signal to protect the ribosome from degradation (left panel). Under conditions of starvation, Ubp3 deubiquitinates the Rpl25, promoting degradation of the ribosome (illustrated by dashed lines). (*b*) Proteaphagy. Ubp3 also plays a role in selective autophagy of the RP (blue) and CP (grey) subunits of the proteasome, a process called proteaphagy. Under conditions of starvation, Ubp3 deubiquitinates the 20S core of the proteasome, preventing its degradation. (*c*) Mitochondrial homeostasis. Regulation of mitochondrial homeostasis by the Ubp3, Ubp2 and Ubp12 DUBs is illustrated. The left panel illustrates deubiquitination of mitochondrial proteins by Ubp3 in complex with cofactor Bre5, preventing autophagic degradation of the mitochondria or mitophagy. The right panel diagrams the role of DUBs Ubp2 and Ubp12 in mitochondrial fission and fusion. Mitochondrial fusion is mediated by mitofusin, Fzo1 (grey stalks), which is ubiquitinated at two locations by distinct E3 ligases. Mdm30 ubiquitinates and stabilizes mitofusion (green Ub chain) thus promoting mitochondrial fusion. Ubp12 can remove these activating Ub chains and promote mitochondrial fragmentation. An unknown E3 ligase can ubiquitinate mitofusion at a distinct site (red Ub chain), and this modification can promote mitofusin degradation. Ubp2 can remove this destabilizing Ub chain and thus promote mitochondrial fusion. (*d*) Transcriptional regulation. Shown is a model for nucleosome stability in yeast regulated by the DUB Ubp8. The nucleosome on the left carries a Ub modification on its histone H2B subunit, which prevents eviction of H2A–H2B, stabilizing the nucleosome and repressing transcription. The nucleosome on the right is rendered unstable owing to the removal of Ub by Ubp8, leading to its disassembly by histone chaperones and/or other regulatory complexes, and activation of transcription by RNAPII (RNA PolII).

Ribophagy is an autophagic mechanism in which the 60S ribosomal subunits are selectively degraded upon nitrogen starvation [121]. In normal conditions, the 60S ribosomal subunits are protected from degradation by ubiquitination of the Rpl25 component by the ribosome-associated E3 ligase, Ltn1 [122]. Upon nutrient limitation, the DUB Ubp3 deubiquitinates L25 and targets 60S subunits for degradation via autophagy in a Cdc48-Ufd3-dependent manner [123] (figure 5*a*). Hence, in this context, Ubp3 activity favours degradation of its substrate. Additionally, under conditions of nitrogen starvation, Ubp3 confers selectivity in the degradation of other factors involved in translation (eIF4GI, eRF3) and mRNA degradation (Dcp2, and Pop2) via autophagic and proteasomal degradation, respectively [124]. Therefore, by regulating the fate of both the translational and mRNA-degradation apparatus, Ubp3 seems to play a key role in reprogramming protein expression in response to nutritional stress.

Nitrogen starvation also induces selective autophagy of the both the RP and CP subunits of the proteasomes, a process termed proteaphagy [125,126]. Nitrogen starvation-induced proteaphagy is dependent on Atg1, a serine–threonine kinase and master regulator of autophagy as well as the core autophagy machinery [125,126]. However, degradation of the CP alone seems to be further fine-tuned by Ubp3 [126], placing Ubp3 at the crossroads of two major proteolytic pathways in the cell—proteasome-mediated degradation and autophagy (figure 5*b*). While the mechanistic details remain to be explored, these results highlight the pivotal roles for DUBs in maintaining protein homeostasis.

## 8. Roles for deubiquitinases in regulation of organelle morphology

### 8.1. Mitochondrial homeostasis

Mitochondria form a dynamic network that is constantly remodelled by their fusion and fission. While fusion promotes mixing of mitochondria, protects against loss of mitochondrial DNA and supports an optimal bioenergetic activity, their fission promotes their distribution and inheritance [127,128]. The mitofusin Fzo1 is a conserved dynamin-related GTPase that resides in the mitochondrial outer membrane and mediates the fusion of mitochondria. guanosine triphosphate (GTP)-binding promotes Fzo1 homodimerization and further oligomerization occurs upon tethering of two mitochondria [127]. Subsequent GTP hydrolysis probably triggers a conformational change in Fzo1 allowing initial ubiquitylation of Fzo1 at K464, mediated by the SCF^Mdm30^ E3 ligase [129–132]. This modification induces Ub chain formation on K398 of a neighbouring Fzo1 molecule that further promotes outer membrane fusion by enhancing intermolecular interactions. The DUB Ubp12 deubiquitinates Fzo1 at K398 resulting in reduced oligomerization of Fzo1, thus antagonizing mitochondrial fusion. Fzo1 is degraded by the proteasome through ubiquitination at other lysine residues, whose modifications are antagonized by the deubiquitinating activity of Ubp2. Thus, by acting through two independent pathways, Ubp2 and Ubp12 regulate Fzo1 stability and oligomerization to control mitochondrial fusion and integrity [132] (figure 5*c*).

Recently, a method termed synthetic quantitative array technology was developed to identify modulators of selective autophagic degradation of mitochondria, a process termed mitophagy, on a genome-wide level [133]. The DUB, Ubp3, complexed with its cofactor, Bre5, was identified as a negative regulator of mitophagy. Additionally, the Ubp3–Bre5 complex, which is typically cytosolic, translocates dynamically to mitochondria under conditions that trigger mitophagy (target of rapamycin inhibition by rapamycin treatment) [133–135]. The molecular mechanism by which Ubp3–Bre5 exerts its influence on mitophagy is unknown.

### 8.2. Regulation of COPI and COPII vesicles

Trafficking of proteins and lipids between the ER and Golgi takes place through the COPII and COPI vesicles, respectively. Regulated polymerization of COPII coatomers including Sec23 onto the ER deforms the lipid bilayer and results in formation of COPII-coated vesicles. Likewise, COPI vesicles are formed at the Golgi by polymerization of COPI coatomers that include Sec27 [136]. Both Sec23 and Sec27 are ubiquitinated and targeted to the proteasome by the Rsp5 E3 ligase and the Cdc48 complex, and this activity is antagonized by the Ubp3–Bre5 DUB [137–139]. The biological importance of this DUB activity is illustrated by a massive expansion of the ER seen in *ubp3Δ* and *bre5Δ* mutant cells. Hence, efficient protein trafficking involves a regulatory balancing act between the E3 Rsp5 and the DUB complex, Ubp3–Bre5.

## 9. Deubiquitinase-mediated regulation of chromosome segregation

Cse4 is a centromere-specific histone variant, which contributes to the unique attributes of centromeric chromatin that enable attachment to the mitotic spindle. Mislocalization of Cse4 to regions outside the centromere is deleterious and causes aberrant chromosome behaviour and mitotic loss. Non-kinetochore Cse4 is ubiquitinated by the Psh1 E3 ubiquitin ligase and targeted for degradation via the proteasome. At kinetochores, the stability of Cse4 is modulated by the Ubp8 DUB [140]. In *ubp8Δ* cells, Cse4 accumulates a short oligomeric chain of Ub (that is Psh1 independent) and is rapidly degraded. Moreover, Cse4 seems to be massively mislocalized in *ubp8Δ* cells leading to chromosomal instability [140]. The E3 ligases Ubr1, Slx5 and SCF (through the Rcy1 F-box protein) also regulate the ubiquitination and degradation of Cse4 [141]. How these multiple E3 ligases work with the DUB Ubp8 to ensure appropriate Cse4 degradation remains poorly understood.

## 10. Roles for deubiquitinases in regulation of transcription

### 10.1. Proteolytic regulation of RNA polymerase II

Stalling of RNA polymerase II (RNAPII) transcription causes polyubiquitination of Rpb1, the largest subunit of RNAPII, and its subsequent degradation by the proteasome leading to disassembly of the RNAPII complex and cessation of RNAPII stalling. The machinery used for ubiquitination of Rbp1 depends on whether or not the RNAPII stalling reflects the cellular response to DNA damage [142–149]. RNAPII stalling induced by DNA damage involves association of the Elc1–Cul3 E3 ligase with Def1, an RNAPII degradation factor, to ubiquitinate Rbp1. By contrast, during DNA damage-independent RNAPII stalling, there is an interplay between the Rsp5 and Elc1/Cul3 E3 ligases along with Def1 to ubiquitinate Rpb1 [144]. In addition, the Cdc48/Ufd1/Npl4 complex and its Ub-binding adaptors Ubx5 and Ubx4 are required for ultraviolet-induced DNA damage-dependent degradation of Rbp1 in stalled RNAPII [149]. Whether this group of regulators is also involved in DNA damage-independent degradation of Rbp1 is not known.

In both DNA damage-dependent and -independent cases, Rbp1 is degraded as a ‘last resort’, when RNAPII is persistently stalled. As a first line of defence, the Ubp3 and Ubp6/Ubp2 DUBs [144] deubiquitinate Rbp1 during DNA damage-dependent and -independent RNAPII stalling, respectively, allowing RNAPII to recover from stalling. Also, Ubp2/Ubp6 action helps reverse K63 polyubiquitin chains built on Rbp1 by Rsp5, and instead promotes Elc1/Cul3-mediated K48 polyubiquitination, targeting it for proteasomal degradation. Thus, the DUBs Ubp2/Ubp6 coordinate the ‘tag-teaming’ of two E3 ligases via their Ub chain editing functions to overcome transcriptional stalling of RNAPII [143].

### 10.2. Regulation of RNA polymerase I and transcription factor stability

The stability of Rpa190, the largest subunit of RNAPI, is regulated by Ubp10. Ubp10 deubiquitinates Rpa190 preventing its proteasomal degradation, thus controlling rRNA production and coordinating cell growth [150]. Also, Tbp1, the TATA-binding protein, is essential for transcriptional activation mediated by Gal4 and Gcn4. The Ubp3–Bre5 complex deubiquitinates Tbp1 directly at promoters, antagonizing its proteasomal degradation [151]. Thus, Ubp3 is required for transcriptional activation of Tbp1-dependent genes.

### 10.3. Regulation of chromatin dynamics (histone H2B ubiquitination)

Chromatin plays a crucial role in regulating transcription and Ub modification of histones plays a significant role in chromatin compaction [152,153]. Histone H2B is ubiquitinated at K123 by the Rad6/Bre1 E3 ligase which in turn primes H3 for repressive methylation (H3K4me3 and H3K76me3) via recruitment of the COMPASS and Dot1 histone methylases [154]. Ubp8 and Ubp10 are both histone H2B DUBs [155–157], but they have very different functions. Ubp8 catalyses H2B deubiquitination *in vitro* and loss of Ubp8 increases the global level of H2B ubiquitination *in vivo* [155,156], suggesting that Ubp8 is the major H2B DUB. Ubp8 colocalizes with H3K4me3, while Ubp10 binds to H3K79me3-enriched sites, as well as telomeres and the rDNA locus [157,158]. Furthermore, as a component of the SAGA acetylation complex, Ubp8 is required for the transcription of SAGA-regulated genes [154]. H2B-Ub deubiquitination by Ubp8 promotes Pol II CTD phosphorylation, which is a hallmark of transcription elongation and is required for co-transcriptional mRNA processing [159] (figure 5*d*). Thus, Ubp8 and Ubp10 act as modulators of crosstalk between different PTMs on histones.

## 11. Cell signalling: interplay between deubiquitination and other post-translational modifications

Multiple interconnected signalling networks allow yeast cells to adjust their metabolism, gene expression, mating and developmental programmes in response to internal and external stimuli [160]. DUBs are versatile components of signalling networks in yeast, often influencing the activity of key regulators such as protein kinases. For example, Ubp8 positively regulates signalling by deubiquitinating the AMP-activated protein kinase, Snf1, and promoting its stability [161]. Additionally, via an obscure mechanism, Ubp8 and Ubp10 together influence the activity of Snf1 through modulation of its phosphorylation status [162]. By contrast, deubiquitination of other kinases and their regulators by Ubp3 promotes their proteasomal degradation, causing inhibition of downstream signalling events including: (i) the cell wall integrity pathway kinase Pkc1 during cell wall stress [163]; (ii) the mating activated protein kinase Ste7 [164,165] during the pheromone response and; (iii) the RasGAP, Ira2, that functions in the protein kinase A pathway [166].

The yeast Hog1 protein is a stress-activated protein kinase that physically interacts with and phosphorylates Ubp3 at Ser695. Phosphorylated Ubp3 is recruited to osmo-responsive genes and facilitates efficient initiation of transcription by deubiquitinating Rpb1, the largest subunit of RNAPII [167]. Several additional phosphorylation sites have been identified in other yeast DUBs (summarized in table 1) whose functional relevance remains to be explored. The DUB Ubp2 is modified by oxidation of its catalytic cysteine following exposure to hydrogen peroxide, inactivating its DUB activity. This inactivation leads to accumulation of K63-linked polyubiquitin chains on ribosomal proteins and stabilization of the assembled 80S ribosome and the polysome to help cells cope with oxidative stress [168]. As noted earlier, several DUBs have cysteine as their catalytic residue, thus signalling-based redox regulation of DUBs may be a widespread theme in DUB biology.

## 12. Roles of deubiquitinases in regulation of the cell cycle

Ubiquitination of cell cycle regulators is integral to ensuring appropriate regulation of cell division, and specific roles for DUBs in cell cycle control have been discovered. For example, ubiquitylation of proliferating cell nuclear antigen (PCNA), a sliding clamp protein with many roles in DNA replication, plays a key role in the tolerance to DNA damage in eukaryotes. Ubp10 forms a complex with PCNA resulting in its deubiquitylation during S-phase and loss of *UBP10* differentially alters the interaction of PCNA with DNA polymerase *ζ*-associated protein Rev1 and with an accessory subunit Rev7. Specifically, while mutation of *UBP10* enhances PCNA–Rev1 interaction, it decreases Rev7 binding to the sliding clamp [169]. Additionally, Ubp10 prevents degradation of Dbf4, the regulatory subunit of the Cdc7 kinase complex that initiates DNA replication [62]. The G1/S-phase transition of the cell cycle is also regulated by Ubp15, which stabilizes the S-phase cyclin Clb5, thus promoting entry into S-phase.

## 13. Perspectives

We are beginning to understand the general principles of DUB function, but many questions about the mechanisms of substrate and polyubiquitin chain recognition by DUBs and their condition-specific functions remain to be explored. Also, the extensive PTM of DUBs, including ubiquitination, sumoylation and phosphorylation, suggest that DUBs are regulated by and involved in many signalling pathways in the cell, yet the function of most PTMs on DUBs is unknown. Advances in mass spectrometric methods for detecting PTMs on proteins promise to help advance our understanding of DUB regulation and help in the identification of new DUB targets. Given the high degree of conservation of biological processes between yeast and humans, systematic studies in yeast should provide a reference map for unravelling human DUB function.

## Data accessibility

This article has no additional data.

## References

[1] Komander D 2009 The emerging complexity of protein ubiquitination. Biochem. Soc. Trans. 37(Pt 5), 937–953. (10.1042/BST0370937) 19754430

[2] Capili AD, Lima CD 2007 Taking it step by step: mechanistic insights from structural studies of ubiquitin/ubiquitin-like protein modification pathways. Curr. Opin. Struct. Biol. 17, 726–735. (10.1016/j.sbi.2007.08.018) 17919899 PMC2174906

[3] Komander D, Clague MJ, Urbe S 2009 Breaking the chains: structure and function of the deubiquitinases. Nat. Rev. Mol. Cell Biol. 10, 550–563. (10.1038/nrm2731) 19626045

[4] Li W, Ye Y 2008 Polyubiquitin chains: functions, structures, and mechanisms. Cell Mol. Life Sci. 65, 2397–2406. (10.1007/s00018-008-8090-6) 18438605 PMC2700825

[5] Nijman SM, Luna-Vargas MPA, Velds A, Brummelkamp TR, Dirac AMG, Sixma TK, Bernards R. 2005 A genomic and functional inventory of deubiquitinating enzymes. Cell 123, 773–786. (10.1016/j.cell.2005.11.007) 16325574

[6] Clague MJ, Barsukov I, Coulson JM, Liu H, Rigden DJ, Urbé S 2013 Deubiquitylases from genes to organism. Physiol. Rev. 93, 1289–1315. (10.1152/physrev.00002.2013) 23899565

[7] He M, Zhou Z, Shah AA, Zou H, Tao J, Chen Q, Wan Y 2016 The emerging role of deubiquitinating enzymes in genomic integrity, diseases, and therapeutics. Cell Biosci. 6, 62 (10.1186/s13578-016-0127-1) 28031783 PMC5168870

[8] Heideker J, Wertz IE 2015 DUBs, the regulation of cell identity and disease. Biochem. J. 465, 1–26. (10.1042/BJ20140496) 25631680

[9] Hussain S, Zhang Y, Galardy PJ 2009 DUBs and cancer: the role of deubiquitinating enzymes as oncogenes, non-oncogenes and tumor suppressors. Cell Cycle 8, 1688–1697. (10.4161/cc.8.11.8739) 19448430

[10] Botstein D, Fink GR 2011 Yeast: an experimental organism for 21st century biology. Genetics 189, 695–704. (10.1534/genetics.111.130765) 22084421 PMC3213361

[11] Clague MJ, Urbe S, Komander D 2019 Breaking the chains: deubiquitylating enzyme specificity begets function. Nat. Rev. Mol. Cell Biol. 20, 338–352. (10.1038/s41580-019-0099-1) 30733604

[12] Haahr P, Borgermann N, Guo X, Typas D, Achuthankutty D, Hoffmann S, Shearer R, Sixma TK, Mailand N 2018 ZUFSP deubiquitylates K63-linked polyubiquitin chains to promote genome stability. Mol. Cell 70, 165–174.e6. (10.1016/j.molcel.2018.02.024) 29576528

[13] Kwasna D et al. 2018 Discovery and characterization of ZUFSP/ZUP1, a distinct deubiquitinase class important for genome stability. Mol. Cell 70, 150–164e6. (10.1016/j.molcel.2018.02.023) 29576527 PMC5896202

[14] Johnston SC, Larsen CN, Cook WJ, Wilkinson KD, Hill CP 1997 Crystal structure of a deubiquitinating enzyme (human UCH-L3) at 1.8 A resolution. EMBO J. 16, 3787–3796. (10.1093/emboj/16.13.3787) 9233788 PMC1170002

[15] Larsen CN, Krantz BA, Wilkinson KD 1998 Substrate specificity of deubiquitinating enzymes: ubiquitin C-terminal hydrolases. Biochemistry 37, 3358–3368. (10.1021/bi972274d) 9521656

[16] Wilkinson KD, Laleli-Sahin E, Urbauer J, Larsen CN, Shih GH, Haas AL, Walsh ST, Wand AJ 1999 The binding site for UCH-L3 on ubiquitin: mutagenesis and NMR studies on the complex between ubiquitin and UCH-L3. J. Mol. Biol. 291, 1067–1077. (10.1006/jmbi.1999.3038) 10518943

[17] Linghu B, Callis J, Goebl MG 2002 Rub1p processing by Yuh1p is required for wild-type levels of Rub1p conjugation to Cdc53p. Eukaryot. Cell 1, 491–494. (10.1128/EC.1.3.491-494.2002) 12455997 PMC118023

[18] Swaney DL, Beltrao P, Starita L, Guo A, Rush J, Fields S, Krogan NJ, Villen J 2013 Global analysis of phosphorylation and ubiquitylation cross-talk in protein degradation. Nat Methods 10, 676–682. (10.1038/nmeth.2519) 23749301 PMC3868471

[19] Albuquerque CP, Smolka MC, Payne SH, Bafna V, Eng J, Zhou H A multidimensional chromatography technology for in-depth phosphoproteome analysis. Mol. Cell Proteomics 7, 1389–1396. (10.1074/mcp.M700468-MCP200) PMC2493382 18407956

[20] Holt LJ, Tuch BB, Villen J, Johnson AD, Gygi SP, Morgan DO 2009 Global analysis of Cdk1 substrate phosphorylation sites provides insights into evolution. Science 325, 1682–1686. (10.1126/science.1172867) 19779198 PMC2813701

[21] Soulard A, Cremonesi A, Moes S, Schutz F, Jeno P, Hall MN 2010 The rapamycin-sensitive phosphoproteome reveals that TOR controls protein kinase A toward some but not all substrates. Mol. Biol. Cell 21, 3475–3486. (10.1091/mbc.E10-03-0182) 20702584 PMC2947482

[22] Jones MH, Keck JM, Wong CCL, Xu T, Yates JR III, Wilney M 2011 Cell cycle phosphorylation of mitotic exit network (MEN) proteins. Cell Cycle 10, 3435–3440. (10.4161/cc.10.20.17790) 22031224 PMC3266174

[23] Weinert BT, Scholz C, Wagner SA, Iesmantavicius V, Su D, Daniel JA, Choudhary C 2013 Lysine succinylation is a frequently occurring modification in prokaryotes and eukaryotes and extensively overlaps with acetylation. Cell Rep. 4, 42–51. (10.1016/j.celrep.2013.07.024) 23954790

[24] Wu R, Haas W, Dephoure N, Huttlin EL, Zhai B, Sowa ME, Gygi SP 2011 A large-scale method to measure absolute protein phosphorylation stoichiometries. Nat. Methods 8, 677–683. (10.1038/nmeth.1636) 21725298 PMC3146562

[25] Hu M, Li P, Li M, Li W, Yao T, Wu J-W, Gu W, Cohen RE, Shi Y 2002 Crystal structure of a UBP-family deubiquitinating enzyme in isolation and in complex with ubiquitin aldehyde. Cell 111, 1041–1054. (10.1016/S0092-8674(02)01199-6) 12507430

[26] Hu M, Li P, Song L, Jeffrey PD, Chenova TA, Wilkinson KD, Cohen RE, Shi Y 2005 Structure and mechanisms of the proteasome-associated deubiquitinating enzyme USP14. EMBO J. 24, 3747–3756. (10.1038/sj.emboj.7600832) 16211010 PMC1276716

[27] Du J, Fu L, Sui Y, Zhang L 2019 The function and regulation of OTU deubiquitinases. Front. Med. (10.1007/S11684-019-0734-4) 31884527

[28] Mevissen TE et al. 2013 OTU deubiquitinases reveal mechanisms of linkage specificity and enable ubiquitin chain restriction analysis. Cell 154, 169–184. (10.1016/j.cell.2013.05.046) 23827681 PMC3705208

[29] Wang T et al. 2009 Evidence for bidentate substrate binding as the basis for the K48 linkage specificity of otubain 1. J. Mol. Biol. 386, 1011–1023. (10.1016/j.jmb.2008.12.085) 19211026 PMC2682458

[30] Rehman SA, Kristariyanto YA, Choi SY, Nkosi PJ, Weidlich S, Labib K, Hofmann K, Kulathu Y 2016 MINDY-1 is a member of an evolutionarily conserved and structurally distinct new family of deubiquitinating enzymes. Mol. Cell 63, 146–155. (10.1016/j.molcel.2016.05.009) 27292798 PMC4942677

[31] Worden EJ, Padovani C, Martin A 2014 Structure of the Rpn11-Rpn8 dimer reveals mechanisms of substrate deubiquitination during proteasomal degradation. Nat. Struct. Mol. Biol. 21, 220–227. (10.1038/nsmb.2771) 24463465

[32] Dambacher CM, Worden EJ, Herzik MA, Martin A, Lander GC 2016 Atomic structure of the 26S proteasome lid reveals the mechanism of deubiquitinase inhibition. eLife 5, e13027 (10.7554/eLife.13027) 26744777 PMC4749569

[33] Komander D, Randow F 2017 Strange new world: bacteria catalyze ubiquitylation via ADP ribosylation. Cell Host Microbe 21, 127–129. (10.1016/j.chom.2017.01.014) 28182945

[34] Mevissen TET, Komander D 2017 Mechanisms of deubiquitinase specificity and regulation. Annu. Rev. Biochem. 86, 159–192. (10.1146/annurev-biochem-061516-044916) 28498721

[35] Trempe JF 2011 Reading the ubiquitin postal code. Curr. Opin. Struct. Biol. 21, 792–801. (10.1016/j.sbi.2011.09.009) 22036065

[36] Klockner C, Schneider M, Lutz S, Jani D, Kressler D, Stewart M, Hurt E, Köhler A 2009 Mutational uncoupling of the role of Sus1 in nuclear pore complex targeting of an mRNA export complex and histone H2B deubiquitination. J. Biol. Chem. 284, 12 049–12 056. (10.1074/jbc.M900502200) PMC2673274 19269973

[37] Morgan MT, Haj-Yahya M, Ringel AE, Bandi P, Brik A, Wolberger C 2016 Structural basis for histone H2B deubiquitination by the SAGA DUB module. Science 351, 725–728. (10.1126/science.aac5681) 26912860 PMC4863942

[38] Schaefer JB, Morgan DO 2011 Protein-linked ubiquitin chain structure restricts activity of deubiquitinating enzymes. J. Biol. Chem. 286, 45 186–45 196. (10.1074/jbc.M111.310094) PMC3247991 22072716

[39] Turco E, Gallego LD, Schneider M, Köhler A. 2015 Monoubiquitination of histone H2B is intrinsic to the Bre1 RING domain-Rad6 interaction and augmented by a second Rad6-binding site on Bre1. J. Biol. Chem. 290, 5298–5310. (10.1074/jbc.M114.626788) 25548288 PMC4342449

[40] Davies CW, Paul LN, Kim M-I, Das C 2011 Structural and thermodynamic comparison of the catalytic domain of AMSH and AMSH-LP: nearly identical fold but different stability. J. Mol. Biol. 413, 416–429. (10.1016/j.jmb.2011.08.029) 21888914 PMC3321355

[41] Amerik A, Swaminathan S, Krantz BA, Wilkinson KD, Hochstrasser M 1997 *In vivo* disassembly of free polyubiquitin chains by yeast Ubp14 modulates rates of protein degradation by the proteasome. EMBO J. 16, 4826–4838. (10.1093/emboj/16.16.4826) 9305625 PMC1170118

[42] Dayal S, Sparks A, Jacob J, Allende-Vega N, Lane DP, Saville MK 2009 Suppression of the deubiquitinating enzyme USP5 causes the accumulation of unanchored polyubiquitin and the activation of p53. J. Biol. Chem. 284, 5030–5041. (10.1074/jbc.M805871200) 19098288 PMC2696100

[43] Doelling JH, Yan N, Kurepa J, Walker J, Vierstra RD 2001 The ubiquitin-specific protease UBP14 is essential for early embryo development in *Arabidopsis thaliana*. Plant J. 27, 393–405. (10.1046/j.1365-313X.2001.01106.x) 11576424

[44] Reyes-Turcu FE, Ventii KH, Wilkinson KD 2009 Regulation and cellular roles of ubiquitin-specific deubiquitinating enzymes. Annu. Rev. Biochem. 78, 363–397. (10.1146/annurev.biochem.78.082307.091526) 19489724 PMC2734102

[45] Reyes-Turcu FE, Shanks JR, Komander D, Wilkinson KD 2008 Recognition of polyubiquitin isoforms by the multiple ubiquitin binding modules of isopeptidase T. J. Biol. Chem. 283, 19 581–19 592. (10.1074/jbc.M800947200) PMC2443676 18482987

[46] Kristariyanto YA, Abdul Rehman SA, Weidlich S, Knebel A, Kulathu Y 2017 A single MIU motif of MINDY-1 recognizes K48-linked polyubiquitin chains. EMBO Rep. 18, 392–402. (10.15252/embr.201643205) 28082312 PMC5331195

[47] Messick TE, Russell NS, Iwata AJ, Sarachan KL, Shiekhattar R, Shanks JR, Reyes-Turcu FE, Wilkinson KD, Marmorstein R 2008 Structural basis for ubiquitin recognition by the Otu1 ovarian tumor domain protein. J. Biol. Chem. 283, 11 038–11 049. (10.1074/jbc.M704398200) PMC2447653 18270205

[48] Glickman MH, Rubin DM, Fried VA, Finley D 1998 The regulatory particle of the *Saccharomyces cerevisiae* proteasome. Mol. Cell Biol. 18, 3149–3162. (10.1128/MCB.18.6.3149) 9584156 PMC108897

[49] Groll M, Ditzel L, Löwe J, Stock D, Bochtler M, Bartunik HD, Huber R 1997 Structure of 20S proteasome from yeast at 2.4 A resolution. Nature 386, 463–471. (10.1038/386463a0) 9087403

[50] Pickart CM, Cohen RE 2004 Proteasomes and their kin: proteases in the machine age. Nat. Rev. Mol. Cell Biol. 5, 177–187. (10.1038/nrm1336) 14990998

[51] Finley D 2009 Recognition and processing of ubiquitin-protein conjugates by the proteasome. Annu. Rev. Biochem. 78, 477–513. 19489727 10.1146/annurev.biochem.78.081507.101607 PMC3431160

[52] Chernova TA, Allen KD, Wesoloski LM, Shanks JR, Chernoff YO, Wilkinson KD 2003 Pleiotropic effects of Ubp6 loss on drug sensitivities and yeast prion are due to depletion of the free ubiquitin pool. J. Biol. Chem. 278, 52 102–52 115. 10.1074/jbc.M310283200 14559899

[53] Guterman A, Glickman MH 2004 Complementary roles for Rpn11 and Ubp6 in deubiquitination and proteolysis by the proteasome. J. Biol. Chem. 279, 1729–1738. 14581483 10.1074/jbc.M307050200

[54] Leggett DS, Hanna J, Borodovsky A, Crosas B, Schmidt M, Baker RT, Walz T, Ploegh H, Finley D 2002 Multiple associated proteins regulate proteasome structure and function. Mol. Cell 10, 495–507. 12408819 10.1016/s1097-2765(02)00638-x

[55] Maytal-Kivity V, Reis N, Hofmann K, Glickman MH 2002 MPN+, a putative catalytic motif found in a subset of MPN domain proteins from eukaryotes and prokaryotes, is critical for Rpn11 function. BMC Biochem. 3, 28 (10.1186/1471-2091-3-28) 12370088 PMC129983

[56] Verma R, Aravind L, Oania R, McDonald WH, Yates JR, Koonin EV, Deshaies RJ 2002 Role of Rpn11 metalloprotease in deubiquitination and degradation by the 26S proteasome. Science 298, 611–615. (10.1126/science.1075898) 12183636

[57] Yao T, Cohen RE 2002 A cryptic protease couples deubiquitination and degradation by the proteasome. Nature 419, 403–407. (10.1038/nature01071) 12353037

[58] Bashore C, Dambacher CM, Goodall EA, Matyskiela ME, Lander GC, Martin A 2015 Ubp6 deubiquitinase controls conformational dynamics and substrate degradation of the 26S proteasome. Nat. Struct. Mol. Biol. 22, 712–719. (10.1038/nsmb.3075) 26301997 PMC4560640

[59] Lee BH et al. 2016 USP14 deubiquitinates proteasome-bound substrates that are ubiquitinated at multiple sites. Nature 532, 398–401. (10.1038/nature17433) 27074503 PMC4844788

[60] Hanna J et al. 2006 Deubiquitinating enzyme Ubp6 functions noncatalytically to delay proteasomal degradation. Cell 127, 99–111. (10.1016/j.cell.2006.07.038) 17018280

[61] Lee BH et al. 2010 Enhancement of proteasome activity by a small-molecule inhibitor of USP14. Nature 467, 179–184. (10.1038/nature09299) 20829789 PMC2939003

[62] Mapa CE, Arsenault HE, Conti MM, Poti KE, Solomon MJ 2018 A balance of deubiquitinating enzymes controls cell cycle entry. Mol. Biol. Cell 29, 2821–2834. (10.1091/mbc.E18-07-0425) 30207830 PMC6249862

[63] Peth A, Besche HC, Goldberg AL 2009 Ubiquitinated proteins activate the proteasome by binding to Usp14/Ubp6, which causes 20S gate opening. Mol. Cell 36, 794–804. (10.1016/j.molcel.2009.11.015) 20005843 PMC2796264

[64] Peth A, Kukushkin N, Bossé M, Goldberg AL 2013 Ubiquitinated proteins activate the proteasomal ATPases by binding to Usp14 or Uch37 homologs. J. Biol. Chem. 288, 7781–7790. (10.1074/jbc.M112.441907) 23341450 PMC3597817

[65] Chandra A, Chen L, Liang H, Madura K 2010 Proteasome assembly influences interaction with ubiquitinated proteins and shuttle factors. J. Biol. Chem. 285, 8330–8339. (10.1074/jbc.M109.076786) 20061387 PMC2832983

[66] Sakata E, Stengel F, Fukunaga K, Zhou M, Saeki Y, Förster F, Baumeister W, Tanaka K, Robinson CV 2011 The catalytic activity of Ubp6 enhances maturation of the proteasomal regulatory particle. Mol. Cell 42, 637–649. (10.1016/j.molcel.2011.04.021) 21658604

[67] Murata S, Yashiroda H, Tanaka K 2009 Molecular mechanisms of proteasome assembly. Nat. Rev. Mol. Cell Biol. 10, 104–115. (10.1038/nrm2630) 19165213

[68] Doherty GJ, McMahon HT 2009 Mechanisms of endocytosis. Annu. Rev. Biochem. 78, 857–902. (10.1146/annurev.biochem.78.081307.110540) 19317650

[69] Kaksonen M, Sun Y, Drubin DG 2003 A pathway for association of receptors, adaptors, and actin during endocytic internalization. Cell 115, 475–487. (10.1016/S0092-8674(03)00883-3) 14622601

[70] Kaksonen M, Toret CP, Drubin DG 2005 A modular design for the clathrin- and actin-mediated endocytosis machinery. Cell 123, 305–320. (10.1016/j.cell.2005.09.024) 16239147

[71] Reider A, Wendland B 2011 Endocytic adaptors–social networking at the plasma membrane. J. Cell Sci. 124(Pt 10), 1613–1622. (10.1242/jcs.073395) 21536832 PMC3085434

[72] Dores MR, Schnell JD, Maldonado-Baez L, Wendland B, Hicke L 2010 The function of yeast epsin and Ede1 ubiquitin-binding domains during receptor internalization. Traffic 11, 151–160. (10.1111/j.1600-0854.2009.01003.x) 19903324 PMC2896001

[73] Stamenova SD, Dunn R, Adler AS, Hicke L 2004 The Rsp5 ubiquitin ligase binds to and ubiquitinates members of the yeast CIN85-endophilin complex, Sla1-Rvs167. J. Biol. Chem. 279, 16 017–16 025. (10.1074/jbc.M313479200) 14761940

[74] Gupta R, Kus B, Fladd C, Wasmuth J, Tonikian R, Sidhu S, Krogan NJ, Parkinson J, Rotin D 2007 Ubiquitination screen using protein microarrays for comprehensive identification of Rsp5 substrates in yeast. Mol. Syst. Biol. 3, 116 (10.1038/msb4100159) 17551511 PMC1911201

[75] Ziv I et al. 2011 A perturbed ubiquitin landscape distinguishes between ubiquitin in trafficking and in proteolysis. Mol. Cell Proteomics 10, M111 009753 (10.1074/mcp.M111.009753) PMC3098606 21427232

[76] Rotin D, Staub O, Haguenauer-Tsapis R 2000 Ubiquitination and endocytosis of plasma membrane proteins: role of Nedd4/Rsp5p family of ubiquitin-protein ligases. J. Membr. Biol. 176, 1–17. (10.1007/s00232001079) 10882424

[77] Weinberg JS, Drubin DG 2014 Regulation of clathrin-mediated endocytosis by dynamic ubiquitination and deubiquitination. Curr. Biol. 24, 951–959. (10.1016/j.cub.2014.03.038) 24746795 PMC4020423

[78] Zhao Y, MacGurn JA, Liu M, Emr S 2013 The ART-Rsp5 ubiquitin ligase network comprises a plasma membrane quality control system that protects yeast cells from proteotoxic stress. eLife 2, e00459. 23599894 10.7554/eLife.00459 PMC3628405

[79] Wang S, Thibault G, Ng DT 2011 Routing misfolded proteins through the multivesicular body (MVB) pathway protects against proteotoxicity. J. Biol. Chem. 286, 29 376–29 387. (10.1074/jbc.M111.233346) PMC3190743 21708947

[80] Lin CH, Macgurn JA, Chu T, Stefan CJ, Emr SD 2008 Arrestin-related ubiquitin-ligase adaptors regulate endocytosis and protein turnover at the cell surface. Cell 135, 714–725. (10.1016/j.cell.2008.09.025) 18976803

[81] Kee Y, Lyon N, Huibregtse JM 2005 The Rsp5 ubiquitin ligase is coupled to and antagonized by the Ubp2 deubiquitinating enzyme. EMBO J. 24, 2414–2424. (10.1038/sj.emboj.7600710) 15933713 PMC1173151

[82] Lam MH, Emili A 2013 Ubp2 regulates Rsp5 ubiquitination activity *in vivo* and *in vitro*. PLoS ONE 8, e75372 (10.1371/journal.pone.0075372) 24069405 PMC3777918

[83] Kee Y, Muñoz W, Lyon N, Huibregtse JM 2006 The deubiquitinating enzyme Ubp2 modulates Rsp5-dependent Lys63-linked polyubiquitin conjugates in *Saccharomyces cerevisiae*. J. Biol. Chem. 281, 36 724–36 731. (10.1074/jbc.M608756200) 17028178

[84] Lam MH, Urban-Grimal D, Bugnicourt A, Greenblatt JF, Haguenauer-Tsapis R, Emili A. 2009 Interaction of the deubiquitinating enzyme Ubp2 and the e3 ligase Rsp5 is required for transporter/receptor sorting in the multivesicular body pathway. PLoS ONE 4, e4259 (10.1371/journal.pone.0004259) 19165343 PMC2626285

[85] MacGurn JA, Hsu PC, Emr SD 2012 Ubiquitin and membrane protein turnover: from cradle to grave. Annu. Rev. Biochem. 81, 231–259. (10.1146/annurev-biochem-060210-093619) 22404628

[86] Amerik A, Sindhi N, Hochstrasser M 2006 A conserved late endosome-targeting signal required for Doa4 deubiquitylating enzyme function. J. Cell Biol. 175, 825–835. (10.1083/jcb.200605134) 17145966 PMC2064681

[87] Kimura Y, Kawawaki J, Kakiyama Y, Shimoda A, Tanaka K 2014 The ESCRT-III adaptor protein Bro1 controls functions of regulator for free ubiquitin chains 1 (Rfu1) in ubiquitin homeostasis. J. Biol. Chem. 289, 21 760–21 769. (10.1074/jbc.M114.550871) PMC4118134 24962567

[88] Pashkova N, Gakhar L, Winistorfer SC, Sunshine AB, Rich M, Dunham MJ, Yu L, Piper RC 2013 The yeast Alix homolog Bro1 functions as a ubiquitin receptor for protein sorting into multivesicular endosomes. Dev. Cell 25, 520–533. (10.1016/j.devcel.2013.04.007) 23726974 PMC3755756

[89] Richter C, West M, Odorizzi G 2007 Dual mechanisms specify Doa4-mediated deubiquitination at multivesicular bodies. EMBO J. 26, 2454–2464. (10.1038/sj.emboj.7601692) 17446860 PMC1868904

[90] Kimura Y, Tanaka K 2010 Regulatory mechanisms involved in the control of ubiquitin homeostasis. J. Biochem. 147, 793–798. (10.1093/jb/mvq044) 20418328

[91] Papa FR, Amerik AY, Hochstrasser M 1999 Interaction of the Doa4 deubiquitinating enzyme with the yeast 26S proteasome. Mol. Biol. Cell 10, 741–756. (10.1091/mbc.10.3.741) 10069815 PMC25199

[92] Clague MJ, Heride C, Urbe S 2015 The demographics of the ubiquitin system. Trends Cell Biol. 25, 417–426. (10.1016/j.tcb.2015.03.002) 25906909

[93] Hanna J, Leggett DS, Finley D 2003 Ubiquitin depletion as a key mediator of toxicity by translational inhibitors. Mol. Cell Biol. 23, 9251–9261. (10.1128/MCB.23.24.9251-9261.2003) 14645527 PMC309641

[94] Hanna J, Meides A, Zhang DP, Finley D 2007 A ubiquitin stress response induces altered proteasome composition. Cell 129, 747–759. (10.1016/j.cell.2007.03.042) 17512408

[95] Kimura Y, Yashiroda H, Kudo T, Koitabashi S, Murata S, Kakizuka A, Tanaka K 2009 An inhibitor of a deubiquitinating enzyme regulates ubiquitin homeostasis. Cell 137, 549–559. (10.1016/j.cell.2009.02.028) 19410548

[96] Amerik AY, Li SJ, Hochstrasser M 2000 Analysis of the deubiquitinating enzymes of the yeast *Saccharomyces cerevisiae*. Biol. Chem. 381, 981–992. (10.1515/BC.2000.121) 11076031

[97] Leuenberger P, Ganscha S, Kahraman A, Cappelletti V, Boersema PJ, Von Mering C, Claassen M, Picotti P 2017 Cell-wide analysis of protein thermal unfolding reveals determinants of thermostability. Science 355, eaai7825 (10.1126/science.aai7825) 28232526

[98] Chen B, Retzlaff M, Roos T, Frydman J 2011 Cellular strategies of protein quality control. Cold Spring Harb. Perspect. Biol. 3, a004374 (10.1101/cshperspect.a004374) 21746797 PMC3140689

[99] Miller SB, Mogk A, Bukau B 2015 Spatially organized aggregation of misfolded proteins as cellular stress defense strategy. J. Mol. Biol. 427, 1564–1574. (10.1016/j.jmb.2015.02.006) 25681695

[100] Mogk A, Bukau B 2017 Role of sHsps in organizing cytosolic protein aggregation and disaggregation. Cell Stress Chaperones 22, 493–502. (10.1007/s12192-017-0762-4) 28120291 PMC5465027

[101] Tyedmers J, Mogk A, Bukau B 2010 Cellular strategies for controlling protein aggregation. Nat. Rev. Mol. Cell Biol. 11, 777–788. (10.1038/nrm2993) 20944667

[102] Balchin D, Hayer-Hartl M, Hartl FU 2016 *In vivo* aspects of protein folding and quality control. Science 353, aac4354 (10.1126/science.aac4354) 27365453

[103] Cherkasov V et al. 2015 Systemic control of protein synthesis through sequestration of translation and ribosome biogenesis factors during severe heat stress. FEBS Lett. 589, 3654–3664. (10.1016/j.febslet.2015.10.010) 26484595

[104] Cherkasov V, Hofmann S, Druffel-Augustin S, Mogk A, Tyedmers J, Stoecklin G, Bukau B 2013 Coordination of translational control and protein homeostasis during severe heat stress. Curr. Biol. 23, 2452–2462. (10.1016/j.cub.2013.09.058) 24291094

[105] Wallace EW et al. 2015 Reversible, specific, active aggregates of endogenous proteins assemble upon heat stress. Cell 162, 1286–1298. (10.1016/j.cell.2015.08.041) 26359986 PMC4567705

[106] Kaganovich D, Kopito R, Frydman J 2008 Misfolded proteins partition between two distinct quality control compartments. Nature 454, 1088–1095. (10.1038/nature07195) 18756251 PMC2746971

[107] Miller SB et al. 2015 Compartment-specific aggregases direct distinct nuclear and cytoplasmic aggregate deposition. EMBO J. 34, 778–797. (10.15252/embj.201489524) 25672362 PMC4369314

[108] Gallina I et al. 2015 Cmr1/WDR76 defines a nuclear genotoxic stress body linking genome integrity and protein quality control. Nat. Commun. 6, 6533 (10.1038/ncomms7533) 25817432 PMC4389229

[109] Oling D, Eisele F, Kvint K, Nystrom T 2014 Opposing roles of Ubp3-dependent deubiquitination regulate replicative life span and heat resistance. EMBO J. 33, 747–761. (10.1002/embj.201386822) 24596250 PMC4000091

[110] Nostramo R, Varia SN, Zhang B, Emerson MM, Herman PK 2016 The catalytic activity of the Ubp3 deubiquitinating protease is required for efficient stress granule assembly in *Saccharomyces cerevisiae*. Mol. Cell Biol. 36, 173–183. 26503781 10.1128/MCB.00609-15 PMC4702605

[111] Nostramo R, Herman PK 2016 Deubiquitination and the regulation of stress granule assembly. Curr. Genet. 62, 503–506. (10.1007/s00294-016-0571-9) 26852120 PMC4930382

[112] Fang NN, Chan GT, Zhu M, Comyn SA, Persaud A, Deshaies RJ, Rotin D, Gsponer J, Mayor T 2014 Rsp5/Nedd4 is the main ubiquitin ligase that targets cytosolic misfolded proteins following heat stress. Nat. Cell Biol. 16, 1227–1237. (10.1038/ncb3054) 25344756 PMC5224936

[113] Nathan JA, Tae Kim H, Ting L, Gygi SP, Goldberg AL 2013 Why do cellular proteins linked to K63-polyubiquitin chains not associate with proteasomes? EMBO J. 32, 552–565. (10.1038/emboj.2012.354) 23314748 PMC3579138

[114] Fang NN, Zhu M, Rose A, Wu K-P, Mayor T 2016 Deubiquitinase activity is required for the proteasomal degradation of misfolded cytosolic proteins upon heat-stress. Nat. Commun. 7, 12907 (10.1038/ncomms12907) 27698423 PMC5059457

[115] Ast T, Aviram N, Chuartzman SG, Schuldiner M 2014 A cytosolic degradation pathway, prERAD, monitors pre-inserted secretory pathway proteins. J. Cell Sci. 127, 3017–3023. (10.1242/jcs.144386) 24849653

[116] Schmitz C, Kinner A, Kolling R 2005 The deubiquitinating enzyme Ubp1 affects sorting of the ATP-binding cassette-transporter Ste6 in the endocytic pathway. Mol. Biol. Cell 16, 1319–1329. (10.1091/mbc.e04-05-0425) 15635103 PMC551495

[117] Ruggiano A, Foresti O, Carvalho P 2014 Quality control: ER-associated degradation: protein quality control and beyond. J. Cell Biol. 204, 869–879. (10.1083/jcb.201312042) 24637321 PMC3998802

[118] Zattas D, Hochstrasser M 2015 Ubiquitin-dependent protein degradation at the yeast endoplasmic reticulum and nuclear envelope. Crit. Rev. Biochem. Mol. Biol. 50, 1–17. (10.3109/10409238.2014.959889) 25231236 PMC4359062

[119] Xie W, Ng DT 2010 ERAD substrate recognition in budding yeast. Semin. Cell Dev. Biol. 21, 533–539. (10.1016/j.semcdb.2010.02.007) 20178855

[120] Bodnar NO, Rapoport TA 2017 Molecular mechanism of substrate processing by the Cdc48 ATPase complex. Cell 169, 722–735e9. (10.1016/j.cell.2017.04.020) 28475898 PMC5751438

[121] Kraft C, Deplazes A, Sohrmann M, Peter M 2008 Mature ribosomes are selectively degraded upon starvation by an autophagy pathway requiring the Ubp3p/Bre5p ubiquitin protease. Nat. Cell Biol. 10, 602–610. (10.1038/ncb1723) 18391941

[122] Ossareh-Nazari B, Niño CA, Bengtson MH, Lee JW, Joazeiro CA, Dargemont C 2014 Ubiquitylation by the Ltn1 E3 ligase protects 60S ribosomes from starvation-induced selective autophagy. J. Cell Biol. 204, 909–917. (10.1083/jcb.201308139) 24616224 PMC3998797

[123] Ossareh-Nazari B, Nazari B, Bonizec M, Cohen M, Dokudovskaya S, Delalande F, Schaeffer C, Van Dorsselaer A, Dargemont C. 2010 Cdc48 and Ufd3, new partners of the ubiquitin protease Ubp3, are required for ribophagy. EMBO Rep. 11, 548–554. (10.1038/embor.2010.74) 20508643 PMC2897114

[124] Kelly SP, Bedwell DM 2015 Both the autophagy and proteasomal pathways facilitate the Ubp3p-dependent depletion of a subset of translation and RNA turnover factors during nitrogen starvation in *Saccharomyces cerevisiae*. RNA 21, 898–910. (10.1261/rna.045211.114) 25795416 PMC4408797

[125] Marshall RS, McLoughlin F, Vierstra RD 2016 Autophagic turnover of inactive 26S proteasomes in yeast is directed by the ubiquitin receptor cue5 and the Hsp42 chaperone. Cell Rep. 16, 1717–1732. (10.1016/j.celrep.2016.07.015) 27477278

[126] Waite KA, Mota-Peynado AD-L, Vontz G, Roelofs J 2016 Starvation induces proteasome autophagy with different pathways for core and regulatory particles. J. Biol. Chem. 291, 3239–3253. (10.1074/jbc.M115.699124) 26670610 PMC4751371

[127] Westermann B 2010 Mitochondrial fusion and fission in cell life and death. Nat. Rev. Mol. Cell Biol. 11, 872–884. (10.1038/nrm3013) 21102612

[128] Friedman JR, Nunnari J 2014 Mitochondrial form and function. Nature 505, 335–343. (10.1038/nature12985) 24429632 PMC4075653

[129] Amiott EA, Cohen MM, Saint-Georges Y, Weissman AM, Fox TD 2009 A mutation associated with CMT2A neuropathy causes defects in Fzo1 GTP hydrolysis, ubiquitylation, and protein turnover. Mol. Biol. Cell 20, 5026–5035. (10.1091/mbc.e09-07-0622) 19812251 PMC2785744

[130] Anton F, Fres JM, Schauss A, Pinson B, Praefcke GJK, Langer T, Escobar-Henriques M 2011 Ugo1 and Mdm30 act sequentially during Fzo1-mediated mitochondrial outer membrane fusion. J. Cell Sci. 124, 1126–1135. (10.1242/jcs.073080) 21385840

[131] Cohen MM, Amiott EA, Day AR, Leboucher GP, Pryce EN, Glickman MH, Mccaffery JM, Shaw JM, Weissman AM 2011 Sequential requirements for the GTPase domain of the mitofusin Fzo1 and the ubiquitin ligase SCFMdm30 in mitochondrial outer membrane fusion. J. Cell Sci. 124, 1403–1410. (10.1242/jcs.079293) 21502136 PMC3078809

[132] Anton F, Dittmar G, Langer T, Escobar-Henriques M 2013 Two deubiquitylases act on mitofusin and regulate mitochondrial fusion along independent pathways. Mol. Cell 49, 487–498. (10.1016/j.molcel.2012.12.003) 23317502

[133] Muller M, Kötter P, Behrendt C, Walter E, Scheckhuber CQ, Entian K-D, Reichert AS 2015 Synthetic quantitative array technology identifies the Ubp3-Bre5 deubiquitinase complex as a negative regulator of mitophagy. Cell Rep. 10, 1215–1225. (10.1016/j.celrep.2015.01.044) 25704822

[134] Chong YT, Koh J, Friesen H, Kaluarachchi Duffy S, Cox M, Moses A, Moffat J, Boone C, Andrews B 2015 Yeast proteome dynamics from single cell imaging and automated analysis. Cell 161, 1413–1424. (10.1016/j.cell.2015.04.051) 26046442

[135] Koh JL, Chong YT, Friesen H, Moses A, Boone C, Andrews BJ, Moffat J 2015 CYCLoPs: a comprehensive database constructed from automated analysis of protein abundance and subcellular localization patterns in *Saccharomyces cerevisiae*. G3 (Bethesda) 5, 1223–1232. (10.1534/g3.115.017830) 26048563 PMC4478550

[136] Yorimitsu T, Sato K, Takeuchi M 2014 Molecular mechanisms of Sar/Arf GTPases in vesicular trafficking in yeast and plants. Front. Plant Sci. 5, 411 (10.3389/fpls.2014.00411) 25191334 PMC4140167

[137] Cohen M, Stutz F, Dargemont C 2003 Deubiquitination, a new player in Golgi to endoplasmic reticulum retrograde transport. J. Biol. Chem. 278, 51 989–51 992. (10.1074/jbc.C300451200) 14593109

[138] Cohen M, Stutz F, Belgareh N, Haguenauer-Tsapis R, Dargemont C. 2003 Ubp3 requires a cofactor, Bre5, to specifically de-ubiquitinate the COPII protein, Sec23. Nat. Cell Biol. 5, 661–667. (10.1038/ncb1003) 12778054

[139] Ossareh-Nazari B, Cohen M, Dargemont C 2010 The Rsp5 ubiquitin ligase and the AAA-ATPase Cdc48 control the ubiquitin-mediated degradation of the COPII component Sec23. Exp. Cell Res. 316, 3351–3357. (10.1016/j.yexcr.2010.09.005) 20846524

[140] Canzonetta C, Vernarecci S, Iuliani M, Marracino C, Belloni C, Ballario P, Filetici P 2015 SAGA DUB-Ubp8 deubiquitylates centromeric histone variant Cse4. G3 (Bethesda) 6, 287–298. (10.1534/g3.115.024877) 26613948 PMC4751549

[141] Cheng H, Bao X, Gan X, Luo S, Rao H 2017 Multiple E3 s promote the degradation of histone H3 variant Cse4. Sci. Rep. 7, 8565 (10.1038/s41598-017-08923-w) 28819127 PMC5561092

[142] Beaudenon SL, Huacani MR, Wang G, Mcdonnell DP, Huibregtse JM 1999 Rsp5 ubiquitin-protein ligase mediates DNA damage-induced degradation of the large subunit of RNA polymerase II in *Saccharomyces cerevisiae*. Mol. Cell Biol. 19, 6972–6979. (10.1128/MCB.19.10.6972) 10490634 PMC84692

[143] Harreman M et al. 2009 Distinct ubiquitin ligases act sequentially for RNA polymerase II polyubiquitylation. Proc. Natl Acad. Sci. USA 106, 20 705–20 710. (10.1073/pnas.0907052106) PMC2778569 19920177

[144] Karakasili E, Burkert-Kautzsch C, Kieser A, Sträßer K. 2014 Degradation of DNA damage-independently stalled RNA polymerase II is independent of the E3 ligase Elc1. Nucleic Acids Res. 42, 10 503–10 515. (10.1093/nar/gku731) PMC4176355 25120264

[145] Malik S, Bagla S, Chaurasia P, Duan Z, Bhaumik SR 2008 Elongating RNA polymerase II is disassembled through specific degradation of its largest but not other subunits in response to DNA damage *in vivo*. J. Biol. Chem. 283, 6897–6905. (10.1074/jbc.M707649200) 18195014

[146] Mao P, Meas R, Dorgan KM, Smerdon MJ 2014 UV damage-induced RNA polymerase II stalling stimulates H2B deubiquitylation. Proc. Natl Acad. Sci. USA 111, 12 811–12 816. (10.1073/pnas.1403901111) PMC4156752 25136098

[147] Reid J, Svejstrup JQ 2004 DNA damage-induced Def1-RNA polymerase II interaction and Def1 requirement for polymerase ubiquitylation *in vitro*. J. Biol. Chem. 279, 29 875–29 878. (10.1074/jbc.C400185200) 15166235

[148] Ribar B, Prakash L, Prakash S 2007 ELA1 and CUL3 are required along with ELC1 for RNA polymerase II polyubiquitylation and degradation in DNA-damaged yeast cells. Mol. Cell Biol. 27, 3211–3216. (10.1128/MCB.00091-07) 17296727 PMC1899920

[149] Verma R, Oania R, Fang R, Smith GT, Deshaies RJ 2011 Cdc48/p97 mediates UV-dependent turnover of RNA Pol II. Mol. Cell 41, 82–92. (10.1016/j.molcel.2010.12.017) 21211725 PMC3063307

[150] Richardson LA, Reed BJ, Charette JM, Freed EF, Fredrickson EK, Locke MN, Baserga SJ, Gardner RG 2012 A conserved deubiquitinating enzyme controls cell growth by regulating RNA polymerase I stability. Cell Rep. 2, 372–385. (10.1016/j.celrep.2012.07.009) 22902402 PMC3638920

[151] Chew BS, Siew WL, Xiao B, Lehming N 2010 Transcriptional activation requires protection of the TATA-binding protein Tbp1 by the ubiquitin-specific protease Ubp3. Biochem. J. 431, 391–399. (10.1042/BJ20101152) 20738257

[152] Bannister AJ, Kouzarides T 2011 Regulation of chromatin by histone modifications. Cell Res. 21, 381–395. (10.1038/cr.2011.22) 21321607 PMC3193420

[153] Bowman GD, Poirier MG 2015 Post-translational modifications of histones that influence nucleosome dynamics. Chem. Rev. 115, 2274–2295. (10.1021/cr500350x) 25424540 PMC4375056

[154] Cao J, Yan Q 2012 Histone ubiquitination and deubiquitination in transcription, DNA damage response, and cancer. Front. Oncol. 2, 26. 22649782 10.3389/fonc.2012.00026 PMC3355875

[155] Henry KW et al. 2003 Transcriptional activation via sequential histone H2B ubiquitylation and deubiquitylation, mediated by SAGA-associated Ubp8. Genes Dev. 17, 2648–2663. (10.1101/gad.1144003) 14563679 PMC280615

[156] Daniel JA, Torok MS, Sun Z-W, Schieltz D, Allis CD, Yates JR, Grant PA 2004 Deubiquitination of histone H2B by a yeast acetyltransferase complex regulates transcription. J. Biol. Chem. 279, 1867–1871. (10.1074/jbc.C300494200) 14660634

[157] Emre NC et al. 2005 Maintenance of low histone ubiquitylation by Ubp10 correlates with telomere-proximal Sir2 association and gene silencing. Mol. Cell 17, 585–594. (10.1016/j.molcel.2005.01.007) 15721261

[158] Schulze JM, Hentrich T, Nakanishi S, Gupta A, Emberly E, Shilatifard A, Kobor MS 2011 Splitting the task: Ubp8 and Ubp10 deubiquitinate different cellular pools of H2BK123. Genes Dev. 25, 2242–2247. (10.1101/gad.177220.111) 22056669 PMC3219228

[159] Wyce A et al. 2007 H2B ubiquitylation acts as a barrier to Ctk1 nucleosomal recruitment prior to removal by Ubp8 within a SAGA-related complex. Mol. Cell 27, 275–288. (10.1016/j.molcel.2007.01.035) 17643376

[160] Zaman S, Lippman SI, Zhao X, Broach JR 2008 How *Saccharomyces* responds to nutrients. Annu. Rev. Genet. 42, 27–81. (10.1146/annurev.genet.41.110306.130206) 18303986

[161] Wilson MA, Koutelou E, Hirsch C, Akdemir K, Schibler A, Barton MC, Dent SYR 2011 Ubp8 and SAGA regulate Snf1 AMP kinase activity. Mol. Cell Biol. 31, 3126–3135. (10.1128/MCB.01350-10) 21628526 PMC3147604

[162] Hsu HE, Liu T-N, Yeh C-S, Chang T-H, Lo Y-C, Kao C-F 2015 Feedback control of Snf1 protein and its phosphorylation is necessary for adaptation to environmental stress. J. Biol. Chem. 290, 16 786–16 796. (10.1074/jbc.M115.639443) PMC4505426 25947383

[163] Wang Y, Zhu M, Ayalew M, Ruff JA 2008 Down-regulation of Pkc1-mediated signaling by the deubiquitinating enzyme Ubp3. J. Biol. Chem. 283, 1954–1961. (10.1074/jbc.M705682200) 17986446

[164] Wang Y, Dohlman HG 2002 Pheromone-dependent ubiquitination of the mitogen-activated protein kinase kinase Ste7. J. Biol. Chem. 277, 15 766–15 772. (10.1074/jbc.M111733200) 11864977

[165] Hurst JH, Dohlman HG 2013 Dynamic ubiquitination of the mitogen-activated protein kinase kinase (MAPKK) Ste7 determines mitogen-activated protein kinase (MAPK) specificity. J. Biol. Chem. 288, 18 660–18 671. (10.1074/jbc.M113.475707) PMC3696639 23645675

[166] Li Y, Wang Y 2013 Ras protein/cAMP-dependent protein kinase signaling is negatively regulated by a deubiquitinating enzyme, Ubp3, in yeast. J. Biol. Chem. 288, 11 358–11 365. (10.1074/jbc.M112.449751) PMC3630878 23476013

[167] Sole C, Nadal-Ribelles M, Kraft C, Peter M, Posas F, De Nadal E 2011 Control of Ubp3 ubiquitin protease activity by the Hog1 SAPK modulates transcription upon osmostress. EMBO J. 30, 3274–3284. (10.1038/emboj.2011.227) 21743437 PMC3160652

[168] Silva GM, Finley D, Vogel C 2015 K63 polyubiquitination is a new modulator of the oxidative stress response. Nat. Struct. Mol. Biol. 22, 116–123. (10.1038/nsmb.2955) 25622294 PMC4318705

[169] Gallego-Sanchez A, Andrés S, Conde F, San-Segundo PA, Bueno A 2012 Reversal of PCNA ubiquitylation by Ubp10 in *Saccharomyces cerevisiae*. PLoS Genet. 8, e1002826 (10.1371/journal.pgen.1002826) 22829782 PMC3400564

